# Supplementary material for: Incidence of diabetes following COVID-19 vaccination and SARS-CoV-2 infection in Hong Kong: A population-based cohort study
Source: PLoS Med. 2023 Jul 24;20(7):e1004274. doi: 10.1371/journal.pmed.1004274 (PMC10406181; doi:10.1371/journal.pmed.1004274)
Supplement: S1 Table — (DOCX) [file pmed.1004274.s002.docx]

S1 Table. ICD-9 Clinical Modification (CM) Codes used for disease identification.

| **Diseases** | **ICD-9-CM diagnosis codes** |
| --- | --- |
| Myocardial infarction | 410 |
| Peripheral vascular disease | 441, 443.9, 785.4, V43.4 |
| Cerebrovascular disease | 430-438 |
| Chronic obstructive pulmonary disease | 490-496, 500-505, 506.4 |
| Dementia | 290 |
| Paralysis | 342, 344.1 |
| Chronic renal failure | 582, 585, 586, 588, 583.0-583.2, 583.4, 583.6, 583.7 |
| Mild liver disease | 571.2, 571.4-571.6 |
| Moderate-severe liver disease | 456.0-456.2, 572.2-572.4, 572.8 |
| Ulcers | 531-534 |
| Rheumatoid arthritis and other inflammatory polyarthropathies | 710.0, 710.1, 710.4, 714.0-714.2, 714.81, 725 |
| Malignancy | 140-159, 180-189, 170-172, 174-176, 179, 160-165, 190-195, 200-208 |
| Metastatic solid tumour | 196-199 |
| Mental disorders | 290-299, 300-319 |
| Obesity | 278.0, V85.3, V85.4 |
