# Supplementary material for: Incidence of diabetes following COVID-19 vaccination and SARS-CoV-2 infection in Hong Kong: A population-based cohort study
Source: PLoS Med. 2023 Jul 24;20(7):e1004274. doi: 10.1371/journal.pmed.1004274 (PMC10406181; doi:10.1371/journal.pmed.1004274)
Supplement: S2 Table — (DOCX) [file pmed.1004274.s003.docx]

S2 Table. BNF codes used for medication.

| **Medications** | **BNF code** |
| --- | --- |
| Renin-angiotensin-system agents | 2.5.5 |
| Beta blockers | 2.4 |
| Calcium channel blockers | 2.6.2 |
| Diuretics | 2.2 |
| Nitrates | 2.6.1 |
| Lipid lowering agents | 2.12 |
| Antiarrhythmic drugs | 2.3.2 |
| Cardiac glycosides | 2.1.1 |
| Anti-coagulants | 2.8.1, 2.8.2 |
| Antiplatelets | 2.9 |
| Antifibrinolytics and haemostatics | 2.11 |
| Hormonal therapy | 7.3, 6.4, 8.3 |
| Glucocorticoids | 6.3.2 |
| Antidepressants | 4.3 |
| Non-steroidal anti-inflammatory drugs | 10.1.1 |
| Drugs for gout | 10.1.4 |
| Antiepileptic drugs | 4.8 |
| Antiviral drugs | 5.3 |
| Antibacterial drugs | 5.1 |
| Immunosuppressants | 8.2 |

Note: BNF = British National Formulary
