# Supplementary material for: Incidence of diabetes following COVID-19 vaccination and SARS-CoV-2 infection in Hong Kong: A population-based cohort study
Source: PLoS Med. 2023 Jul 24;20(7):e1004274. doi: 10.1371/journal.pmed.1004274 (PMC10406181; doi:10.1371/journal.pmed.1004274)
Supplement: S3 Table — (DOCX) [file pmed.1004274.s004.docx]

S3 Table. Baseline characteristics of BNT162b2 or CoronaVac recipients, unvaccinated people, COVID-19 patients, and non-COVID-19 people before propensity score matching.

| Baseline characteristics | Unvaccinated (N = 348,403) | CoronaVac (N = 176,099) | SMD | BNT162b2 (N = 182,129) | SMD | COVID-19 Patients (N = 145,452) | Non-COVID-19 People (N = 653,126) | SMD |
| --- | --- | --- | --- | --- | --- | --- | --- | --- |
|  | Mean±SD /N (%) | Mean±SD /N (%) |  | Mean±SD /N (%) |  | Mean±SD /N (%) | Mean±SD /N (%) |  |
| Age, years | 67.9±15.7 | 61.6±12.1 |  | 55.9±14.2 |  | 62.7±16.0 | 61.6±15.1 |  |
| 18-44 | 31,550 (9.1) | 14,586 (8.3) | 0.22 | 39,043 (21.4) | 0.60 | 20,121 (13.8) | 88,198 (13.5) | 0.08 |
| 45-59 | 57,967 (16.6) | 57,338 (32.6) |  | 62,526 (34.3) |  | 37,350 (25.7) | 181,639 (27.8) |  |
| ≥60 | 258,886 (74.3) | 104,175 (59.2) |  | 80,560 (44.2) |  | 87,981 (60.5) | 383,289 (58.7) |  |
| Sex |  |  |  |  |  |  |  |  |
| Male | 152,201 (43.7) | 83,352 (47.3) | 0.07 | 83,650 (45.9) | 0.05 | 67,083 (46.1) | 290,172 (44.4) | 0.03 |
| Female | 196,202 (56.3) | 92,747 (52.7) |  | 98,479 (54.1) |  | 78,369 (53.9) | 362,954 (55.6) |  |
| Previous COVID-19 infection | 818 (0.2) | 459 (0.3) | 0.01 | 1,210 (0.7) | 0.06 | NA | NA | NA |
| Vaccination status |  |  |  |  |  |  |  |  |
| Unvaccinated | 348,403 (100.0) | 0 (0.0) | NA | 0 (0.0) | NA | 25,792 (17.7) | 120,890 (18.5) | 0.08 |
| Partially vaccinated* | 0 (0.0) | 176,099 (100.0) | NA | 182,129 (100.0) | NA | 59,312 (40.8) | 219,329 (33.6) |  |
| Fully vaccinated† | 0 (0.0) | 0 (0.0) | NA | 0 (0.0) | NA | 60,348 (41.5) | 312,907 (47.9) |  |
| Prediabetes status‡ | 173,116 (49.7) | 93,008 (52.9) | 0.06 | 82,640 (45.5) | 0.09 | 70,623 (48.6) | 327,844 (50.2) | 0.03 |
| Pre-existing comorbidities |  |  |  |  |  |  |  |  |
| Charlson Comorbidity Index | 3.5±1.7 | 2.8±1.3 |  | 2.2±1.4 |  | 3.0±1.8 | 2.8±1.7 |  |
| 0 | 21,436 (6.2) | 8,301 (4.7) | 0.26 | 27,507 (15.1) | 0.63 | 13,492 (9.3) | 59,305 (9.1) | 0.02 |
| 1-2 | 63,514 (18.2) | 64,118 (36.4) |  | 74,806 (41.1) |  | 43,332 (29.8) | 206,233 (31.6) |  |
| ≥3 | 263,453 (75.6) | 103,680 (58.9) |  | 79,816 (43.8) |  | 88,628 (60.9) | 387,588 (59.3) |  |
| Myocardial infarction | 7,096 (2.0) | 1,471 (0.8) | 0.10 | 1,095 (0.6) | 0.13 | 2,304 (1.6) | 8,782 (1.3) | 0.02 |
| Peripheral vascular disease | 2,661 (0.8) | 411 (0.2) | 0.08 | 305 (0.2) | 0.09 | 790 (0.5) | 3,031 (0.5) | 0.01 |
| Cerebrovascular disease | 39,162 (11.2) | 8,804 (5.0) | 0.23 | 5,941 (3.3) | 0.31 | 12,455 (8.6) | 48,900 (7.5) | 0.04 |
| Chronic obstructive pulmonary disease | 15,710 (4.5) | 3,896 (2.2) | 0.13 | 3,385 (1.9) | 0.15 | 5,694 (3.9) | 20,209 (3.1) | 0.04 |
| Dementia | 4,233 (1.2) | 367 (0.2) | 0.12 | 126 (0.1) | 0.14 | 1,698 (1.2) | 3,536 (0.5) | 0.07 |
| Paralysis | 2,622 (0.8) | 370 (0.2) | 0.08 | 212 (0.1) | 0.10 | 824 (0.6) | 2,737 (0.4) | 0.02 |
| Chronic renal failure | 9,840 (2.8) | 1,832 (1.0) | 0.13 | 1,387 (0.8) | 0.16 | 3,307 (2.3) | 11,459 (1.8) | 0.04 |
| Mild liver disease | 1,175 (0.3) | 304 (0.2) | 0.03 | 272 (0.1) | 0.04 | 451 (0.3) | 1,616 (0.2) | 0.01 |
| Moderate-severe liver disease | 730 (0.2) | 167 (0.1) | 0.03 | 149 (0.1) | 0.03 | 311 (0.2) | 990 (0.2) | 0.01 |
| Ulcers | 5,934 (1.7) | 1,822 (1.0) | 0.06 | 1,324 (0.7) | 0.09 | 2,209 (1.5) | 8,863 (1.4) | 0.01 |
| Rheumatoid arthritis and other inflammatory polyarthropathies | 4,402 (1.3) | 1,281 (0.7) | 0.05 | 1,541 (0.8) | 0.04 | 1,615 (1.1) | 7,018 (1.1) | 0.00 |
| Malignancy | 15,946 (4.6) | 3,444 (2.0) | 0.15 | 3,323 (1.8) | 0.16 | 5,508 (3.8) | 22,268 (3.4) | 0.02 |
| Metastatic solid tumour | 3,246 (0.9) | 356 (0.2) | 0.10 | 305 (0.2) | 0.10 | 1,063 (0.7) | 3,538 (0.5) | 0.02 |
| Mental disorders | 36,955 (10.6) | 11,295 (6.4) | 0.15 | 10,617 (5.8) | 0.17 | 15,290 (10.5) | 55,159 (8.5) | 0.07 |
| Obesity | 17,019 (4.9) | 10,147 (5.8) | 0.04 | 9,275 (5.1) | 0.01 | 8,475 (5.8) | 33,509 (5.1) | 0.03 |
| Use of medications within 90 days before index date | |  |  |  |  |  |  |  |
| Renin-angiotensin-system agents | 94,390 (27.1) | 36,964 (21.0) | 0.14 | 31,881 (17.5) | 0.23 | 25,230 (17.3) | 107,605 (16.5) | 0.02 |
| Beta blockers | 81,227 (23.3) | 28,306 (16.1) | 0.18 | 23,420 (12.9) | 0.27 | 19,702 (13.5) | 81,962 (12.6) | 0.03 |
| Calcium channel blockers | 143,991 (41.3) | 63,280 (35.9) | 0.11 | 51,333 (28.2) | 0.28 | 41,572 (28.6) | 175,690 (26.9) | 0.04 |
| Diuretics | 33,548 (9.6) | 6,704 (3.8) | 0.23 | 5,351 (2.9) | 0.28 | 7,878 (5.4) | 27,652 (4.2) | 0.06 |
| Nitrates | 28,037 (8.0) | 7,747 (4.4) | 0.15 | 5,962 (3.3) | 0.21 | 5,831 (4.0) | 22,481 (3.4) | 0.03 |
| Lipid lowering agents | 139,176 (39.9) | 58,664 (33.3) | 0.14 | 50,630 (27.8) | 0.26 | 36,655 (25.2) | 165,371 (25.3) | 0.00 |
| Antiarrthymic drugs | 2,732 (0.8) | 602 (0.3) | 0.06 | 591 (0.3) | 0.06 | 641 (0.4) | 2,428 (0.4) | 0.01 |
| Cardiac glycosides | 5,408 (1.6) | 786 (0.4) | 0.11 | 547 (0.3) | 0.13 | 1,166 (0.8) | 3,817 (0.6) | 0.03 |
| Anti-coagulants | 20,220 (5.8) | 3,183 (1.8) | 0.21 | 2,342 (1.3) | 0.25 | 4,500 (3.1) | 16,866 (2.6) | 0.03 |
| Antiplatelets | 90,668 (26.0) | 29,041 (16.5) | 0.23 | 22,082 (12.1) | 0.36 | 21,060 (14.5) | 85,158 (13.0) | 0.04 |
| Antifibrinolytics and haemostatics | 3,285 (0.9) | 1,065 (0.6) | 0.04 | 1,337 (0.7) | 0.02 | 1,056 (0.7) | 3,456 (0.5) | 0.02 |
| Hormonal therapy | 6,909 (2.0) | 2,052 (1.2) | 0.07 | 2,800 (1.5) | 0.03 | 1,638 (1.1) | 5,846 (0.9) | 0.02 |
| Glucocorticoids | 13,225 (3.8) | 2,661 (1.5) | 0.14 | 3,387 (1.9) | 0.12 | 4,006 (2.8) | 13,728 (2.1) | 0.04 |
| Antidepressants | 29,644 (8.5) | 10,773 (6.1) | 0.09 | 11,122 (6.1) | 0.09 | 8,164 (5.6) | 33,078 (5.1) | 0.02 |
| NSAIDs | 23,982 (6.9) | 17,185 (9.8) | 0.10 | 20,009 (11.0) | 0.14 | 9,589 (6.6) | 37,120 (5.7) | 0.04 |
| Drugs for gout | 18,028 (5.2) | 6,223 (3.5) | 0.08 | 5,472 (3.0) | 0.11 | 4,835 (3.3) | 18,511 (2.8) | 0.03 |
| Antiepileptic drugs | 19,609 (5.6) | 5,353 (3.0) | 0.13 | 5,998 (3.3) | 0.11 | 5,627 (3.9) | 20,401 (3.1) | 0.04 |
| Antiviral drugs | 10,495 (3.0) | 5,464 (3.1) | 0.01 | 5,518 (3.0) | 0.00 | 3,999 (2.7) | 12,605 (1.9) | 0.05 |
| Antibacterial drugs | 33,632 (9.7) | 8,249 (4.7) | 0.19 | 8,866 (4.9) | 0.19 | 12,199 (8.4) | 35,554 (5.4) | 0.12 |
| Immunosuppressants | 5,836 (1.7) | 1,449 (0.8) | 0.08 | 2,208 (1.2) | 0.04 | 1,631 (1.1) | 6,853 (1.0) | 0.01 |

Notes: SD = standard deviation; SMD = Standardised mean difference; NSAIDs = Non-steroidal anti-inflammatory drugs; NA = Not applicable

*Partially vaccinated people were defined as vaccine recipients who received one dose of BNT162b2 or no more than two doses of CoronaVac.

†Fully vaccinated people were defined as those with at least two doses of BNT162b2 or three doses of CoronaVac.

‡Prediabetes was defined as baseline HbA1c ≥ 5.7% and < 6.5%.
