# Supplementary material for: Incidence of diabetes following COVID-19 vaccination and SARS-CoV-2 infection in Hong Kong: A population-based cohort study
Source: PLoS Med. 2023 Jul 24;20(7):e1004274. doi: 10.1371/journal.pmed.1004274 (PMC10406181; doi:10.1371/journal.pmed.1004274)
Supplement: S4 Table — (DOCX) [file pmed.1004274.s005.docx]

S4 Table. Crude incidence rate of outcomes for two doses of CoronaVac or BNT162b2 recipients and respective matched controls, and hazard ratio for two doses of CoronaVac or BNT162b2 recipients in comparison with their respective matched controls.

| Events | **Two-dose vaccine recipients** | | | | **Control** | | | | HR† | 95% CI | P-value |
| --- | --- | --- | --- | --- | --- | --- | --- | --- | --- | --- | --- |
|  | Cases with event | Crude incidence rate* | 95% CI | Person-days | Cases with event | Crude incidence rate* | 95% CI | Person-days |  |  |  |
| **CoronaVac recipients vs controls** | | |  |  |  |  |  |  |  |  |  |
| **Overall diabetes** | 3,245 | 10.42 | (10.06, 10.78) | 31,151,363 | 3,421 | 11.12 | (10.75, 11.50) | 30,769,229 | 0.938 | (0.894, 0.984) | 0.009 |
| **Type 2 diabetes** | 3,244 | 10.41 | (10.06, 10.78) | 31,151,377 | 3,420 | 11.11 | (10.75, 11.49) | 30,769,499 | 0.938 | (0.894, 0.984) | 0.009 |
| **Type 1 diabetes** | 1 | 0.00 | (0.00, 0.02) | 31,804,153 | 1 | 0.00 | (0.00, 0.02) | 31,467,889 | 0.980 | (0.063, 15.272) | 0.988 |
|  |  |  |  |  |  |  |  |  |  |  |  |
| **BNT162b2 recipients vs controls** | | |  |  |  |  |  |  |  |  |  |
| **Overall diabetes** | 2,542 | 8.84 | (8.50, 9.19) | 28,762,866 | 2,933 | 10.31 | (9.94, 10.69) | 28,452,855 | 0.857 | (0.813, 0.904) | <0.001 |
| **Type 2 diabetes** | 2,541 | 8.83 | (8.49, 9.18) | 28,763,275 | 2,931 | 10.30 | (9.93, 10.68) | 28,452,987 | 0.857 | (0.813, 0.904) | <0.001 |
| **Type 1 diabetes** | 1 | 0.00 | (0.00, 0.02) | 29,253,997 | 2 | 0.01 | (0.00, 0.02) | 29,049,333 | 0.507 | (0.045, 5.769) | 0.584 |

Notes: HR = Hazard ratio; CI = Confidence interval; NA = Not applicable

*The unit of crude incidence rate: events per 100,000 person-days.

†HR > 1 (or < 1) indicates two-dose vaccine recipients had a higher risk (or lower risk) of outcome compared with their respective matched controls.
