# Supplementary material for: Incidence of diabetes following COVID-19 vaccination and SARS-CoV-2 infection in Hong Kong: A population-based cohort study
Source: PLoS Med. 2023 Jul 24;20(7):e1004274. doi: 10.1371/journal.pmed.1004274 (PMC10406181; doi:10.1371/journal.pmed.1004274)
Supplement: S5 Table — (DOCX) [file pmed.1004274.s006.docx]

S5 Table. Crude incidence rate of outcomes for COVID-19 patients and matched controls, and hazard ratio for COVID-19 patients in comparison with matched controls, censoring at the date of vaccination.

| Events | **COVID-19 patients** | | | | **Control** | | | | HR† | 95% CI | P-value |
| --- | --- | --- | --- | --- | --- | --- | --- | --- | --- | --- | --- |
|  | Cases with event | Crude incidence rate* | 95% CI | Person-days | Cases with event | Crude incidence rate* | 95% CI | Person-days |  |  |  |
| **COVID-19 patients vs controls** | |  |  |  |  |  |  |  |  |  |  |
| **Overall diabetes** | 1,871 | 9.29 | (8.88, 9.72) | 20,132,162 | 879 | 6.87 | (6.43, 7.34) | 12,786,264 | 1.451 | (1.336, 1.575) | <0.001 |
| **Type 2 diabetes** | 1,871 | 9.29 | (8.88, 9.72) | 20,132,162 | 878 | 6.87 | (6.42, 7.34) | 12,786,278 | 1.453 | (1.338, 1.577) | <0.001 |
| **Type 1 diabetes** | 0 | 0.00 | NA | 20,297,141 | 1 | 0.01 | (0.00, 0.04) | 12,848,586 | NA | NA | NA |

Notes: HR = Hazard ratio; CI = Confidence interval; NA = Not applicable

*The unit of crude incidence rate: events per 100,000 person-days.

†HR > 1 (or < 1) indicates COVID-19 patients had a higher risk (or lower risk) of outcome compared with the matched controls.
