# Supplementary material for: Incidence of diabetes following COVID-19 vaccination and SARS-CoV-2 infection in Hong Kong: A population-based cohort study
Source: PLoS Med. 2023 Jul 24;20(7):e1004274. doi: 10.1371/journal.pmed.1004274 (PMC10406181; doi:10.1371/journal.pmed.1004274)
Supplement: S6 Table — (DOCX) [file pmed.1004274.s007.docx]

S6 Table. The restricted mean survival time (RMST) difference and the restricted mean time lost (RMTL) ratio for outcomes.

| Events | RMST difference* (Days) | 95% CI | | P-value | RMTL ratio† | | 95% CI | | | P-value | | Time horizon (Days) | |
| --- | --- | --- | --- | --- | --- | --- | --- | --- | --- | --- | --- | --- | --- |
| **CoronaVac recipients** | | |  |  | |  | |  |  | |  | |  |
| **Overall diabetes** | 0.239 | (-0.236, 0.714) | | 0.323 | 0.982 | | (0.946, 1.018) | | | 0.323 | | 532 | |
| **Type 2 diabetes** | 0.242 | (-0.233, 0.716) | | 0.318 | 0.981 | | (0.946, 1.018) | | | 0.318 | | 532 | |
| **Type 1 diabetes** | -0.002 | (-0.014, 0.01) | | 0.769 | 1.256 | | (0.272, 5.788) | | | 0.770 | | 532 | |
| **BNT162b2 recipients** | | |  |  | |  | |  |  | |  | |  |
| **Overall diabetes** | 1.827 | (1.383, 2.27) | | <0.001 | 0.846 | | (0.812, 0.881) | | | <0.001 | | 521 | |
| **Type 2 diabetes** | 1.822 | (1.379, 2.265) | | <0.001 | 0.846 | | (0.812, 0.881) | | | <0.001 | | 521 | |
| **Type 1 diabetes** | 0.005 | (-0.006, 0.017) | | 0.364 | 0.454 | | (0.082, 2.514) | | | 0.366 | | 522 | |
| **COVID-19 patients** | | |  |  | |  | |  |  | |  | |  |
| **Overall diabetes** | -0.501 | (-0.607, -0.395) | | <0.001 | 1.385 | | (1.293, 1.483) | | | <0.001 | | 189 | |
| **Type 2 diabetes** | -0.501 | (-0.608, -0.395) | | <0.001 | 1.385 | | (1.293, 1.484) | | | <0.001 | | 189 | |
| **Type 1 diabetes** | NA | NA | | NA | NA | | NA | | | NA | | 189 | |

Notes: RMST = Restricted mean survival time; RMTL = Restricted mean time lost; CI = Confidence interval; NA = Not applicable

* RMST difference > 0 (or < 0) indicates vaccine recipients or COVID-19 patients have a longer (or shorter) average of event-free survival time than the matched controls during a specified time horizon.

† RMTL ratio < 1 (or > 1) indicates the vaccine recipients or COVID-19 patients lose less (or more) event-free survival time than the matched controls during the specified time horizon on average.
