# Supplementary material for: Incidence of diabetes following COVID-19 vaccination and SARS-CoV-2 infection in Hong Kong: A population-based cohort study
Source: PLoS Med. 2023 Jul 24;20(7):e1004274. doi: 10.1371/journal.pmed.1004274 (PMC10406181; doi:10.1371/journal.pmed.1004274)
Supplement: S7 Table — (DOCX) [file pmed.1004274.s008.docx]

S7 Table. Crude incidence rate of outcomes for CoronaVac or BNT162b2 recipients, COVID-19 patients, and respective matched controls, and incidence rate ratio of events for CoronaVac or BNT162b2 recipients and COVID-19 patients in comparison with their respective matched controls.

| Events | **Vaccine recipients or COVID-19 patients** | | | | **Control** | | | | IRR† | 95% CI | P-value |
| --- | --- | --- | --- | --- | --- | --- | --- | --- | --- | --- | --- |
|  | Cases with event | Crude incidence rate* | 95% CI | Person-days | Cases with event | Crude incidence rate* | 95% CI | Person-days |  |  |  |
| **CoronaVac recipients vs controls** | |  |  |  |  |  |  |  |  |  |  |
| **Overall diabetes** | 5,760 | 9.08 | (8.85, 9.32) | 63,430,730 | 5,771 | 9.10 | (8.87, 9.34) | 63,384,197 | 0.997 | (0.962, 1.034) | 0.887 |
| **Type 2 diabetes** | 5,756 | 9.07 | (8.84, 9.31) | 63,431,741 | 5,768 | 9.10 | (8.87, 9.34) | 63,384,855 | 0.997 | (0.961, 1.034) | 0.880 |
| **Type 1 diabetes** | 4 | 0.006 | (0.00, 0.02) | 64,660,279 | 3 | 0.00 | (0.00, 0.01) | 64,636,412 | 1.333 | (0.298, 5.955) | 0.707 |
| **BNT162b2 recipients vs controls** | |  |  |  |  |  |  |  |  |  |  |
| **Overall diabetes** | 4,411 | 7.41 | (7.19, 7.63) | 59,532,322 | 5,154 | 8.58 | (8.35, 8.82) | 60,057,014 | 0.863 | (0.829, 0.899) | <0.001 |
| **Type 2 diabetes** | 4,409 | 7.41 | (7.19, 7.63) | 59,532,991 | 5,150 | 8.58 | (8.34, 8.81) | 60,057,786 | 0.864 | (0.830, 0.899) | <0.001 |
| **Type 1 diabetes** | 2 | 0.003 | (0.00, 0.01) | 60,435,717 | 4 | 0.01 | (0.00, 0.02) | 61,173,591 | 0.506 | (0.093, 2.763) | 0.432 |
| **COVID-19 patients vs controls** | |  |  |  |  |  |  |  |  |  |  |
| **Overall diabetes** | 2,109 | 9.04 | (8.66, 9.44) | 23,324,634 | 1,775 | 7.38 | (7.04, 7.73) | 24,050,683 | 1.225 | (1.150, 1.305) | <0.001 |
| **Type 2 diabetes** | 2,109 | 9.04 | (8.66, 9.44) | 23,324,634 | 1,774 | 7.38 | (7.04, 7.73) | 24,050,697 | 1.226 | (1.151, 1.306) | <0.001 |
| **Type 1 diabetes** | 0 | 0.00 | NA | 23,544,532 | 1 | 0.00 | (0.00, 0.02) | 24,208,039 | NA | NA | NA |

Notes: IRR = Incidence rate ratio; CI = Confidence interval; NA = Not applicable

* The unit of crude incidence rate: events per 100,000 person-days.

†IRR > 1 (or < 1) indicates vaccine recipients or COVID-19 patients had a higher risk (or lower risk) of outcome compared with their respective matched controls.
