# Supplementary material for: Incidence of diabetes following COVID-19 vaccination and SARS-CoV-2 infection in Hong Kong: A population-based cohort study
Source: PLoS Med. 2023 Jul 24;20(7):e1004274. doi: 10.1371/journal.pmed.1004274 (PMC10406181; doi:10.1371/journal.pmed.1004274)
Supplement: S8 Table — (DOCX) [file pmed.1004274.s009.docx]

S8 Table. Crude incidence rate of outcomes for CoronaVac or BNT162b2 recipients, unvaccinated people, COVID-19 patients, and non-COVID-19 people before weighting, and hazard ratio after weighting.

| Events | **Before weighting** | | | | **After weighting** | | |
| --- | --- | --- | --- | --- | --- | --- | --- |
|  | Cases with event | Crude incidence rate* | 95% CI | Person-days | HR† | 95%CI | P-value |
| **Overall diabetes** |  |  |  |  |  |  |  |
| **Unvaccinated** | 12,472 | 9.60 | (9.43, 9.77) | 129,911,196 | (reference) | | |
| **BNT16212** | 4,880 | 7.12 | (6.92, 7.32) | 68,520,152 | 0.816 | (0.787, 0.845) | <0.001 |
| **CoronaVac** | 6,063 | 9.08 | (8.85, 9.31) | 66,786,778 | 0.891 | (0.863, 0.919) | <0.001 |
| **Non-COVID-19 people** | 8,092 | 7.47 | (7.31, 7.63) | 108,359,151 | (reference) | | |
| **COVID-19 patients** | 2,113 | 9.04 | (8.66, 9.44) | 23,368,530 | 1.184 | (1.128, 1.243) | <0.001 |
| **Type 2 diabetes** |  |  |  |  |  |  |  |
| **Unvaccinated** | 12,467 | 9.60 | (9.43, 9.77) | 129,912,238 | (reference) | | |
| **BNT16212** | 4,878 | 7.12 | (6.92, 7.32) | 68,520,821 | 0.816 | (0.787, 0.845) | <0.001 |
| **CoronaVac** | 6,059 | 9.07 | (8.85, 9.30) | 66,787,789 | 0.890 | (0.863, 0.919) | <0.001 |
| **Non-COVID-19 people** | 8,090 | 7.47 | (7.30, 7.63) | 108,359,311 | (reference) | | |
| **COVID-19 patients** | 2,113 | 9.04 | (8.66, 9.44) | 23,368,530 | 1.184 | (1.128, 1.243) | <0.001 |
| **Type 1 diabetes** |  |  |  |  |  |  |  |
| **Unvaccinated** | 5 | 0.00 | (0.00, 0.01) | 132,588,451 | (reference) | | |
| **BNT16212** | 2 | 0.00 | (0.00, 0.01) | 69,522,173 | 0.640 | (0.109, 3.743) | 0.620 |
| **CoronaVac** | 4 | 0.01 | (0.00, 0.02) | 68,078,580 | 1.183 | (0.301, 4.651) | 0.810 |
| **Non-COVID-19 people** | 2 | 0.00 | (0.00, 0.01) | 109,109,437 | (reference) | | |
| **COVID-19 patients** | 0 | 0.00 | NA | 23,588,708 | NA | NA | NA |

Notes: HR = Hazard ratio; CI = Confidence interval; NA = Not applicable

*The unit of crude incidence rate: events per 100,000 person-days.

†HR > 1 (or < 1) indicates vaccine recipients or COVID-19 patients had a higher risk (or lower risk) of outcome compared with their respective controls.
