# Supplementary figures and images for: Incidence of diabetes following COVID-19 vaccination and SARS-CoV-2 infection in Hong Kong: A population-based cohort study
Source: PLoS Med. 2023 Jul 24;20(7):e1004274. doi: 10.1371/journal.pmed.1004274 (PMC10406181; doi:10.1371/journal.pmed.1004274)

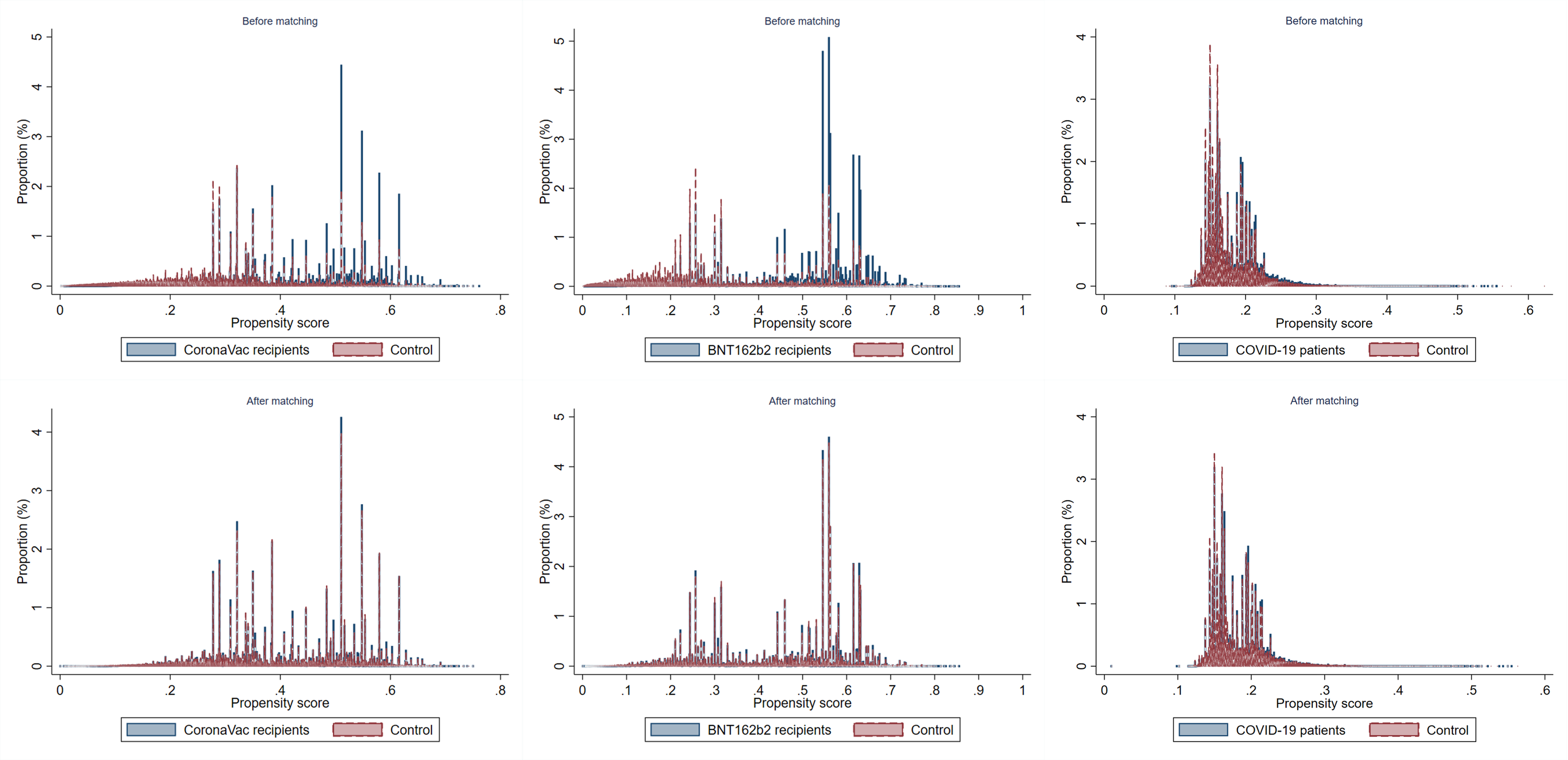

Supplement: S1 Fig — (TIF) [file pmed.1004274.s010.tif]

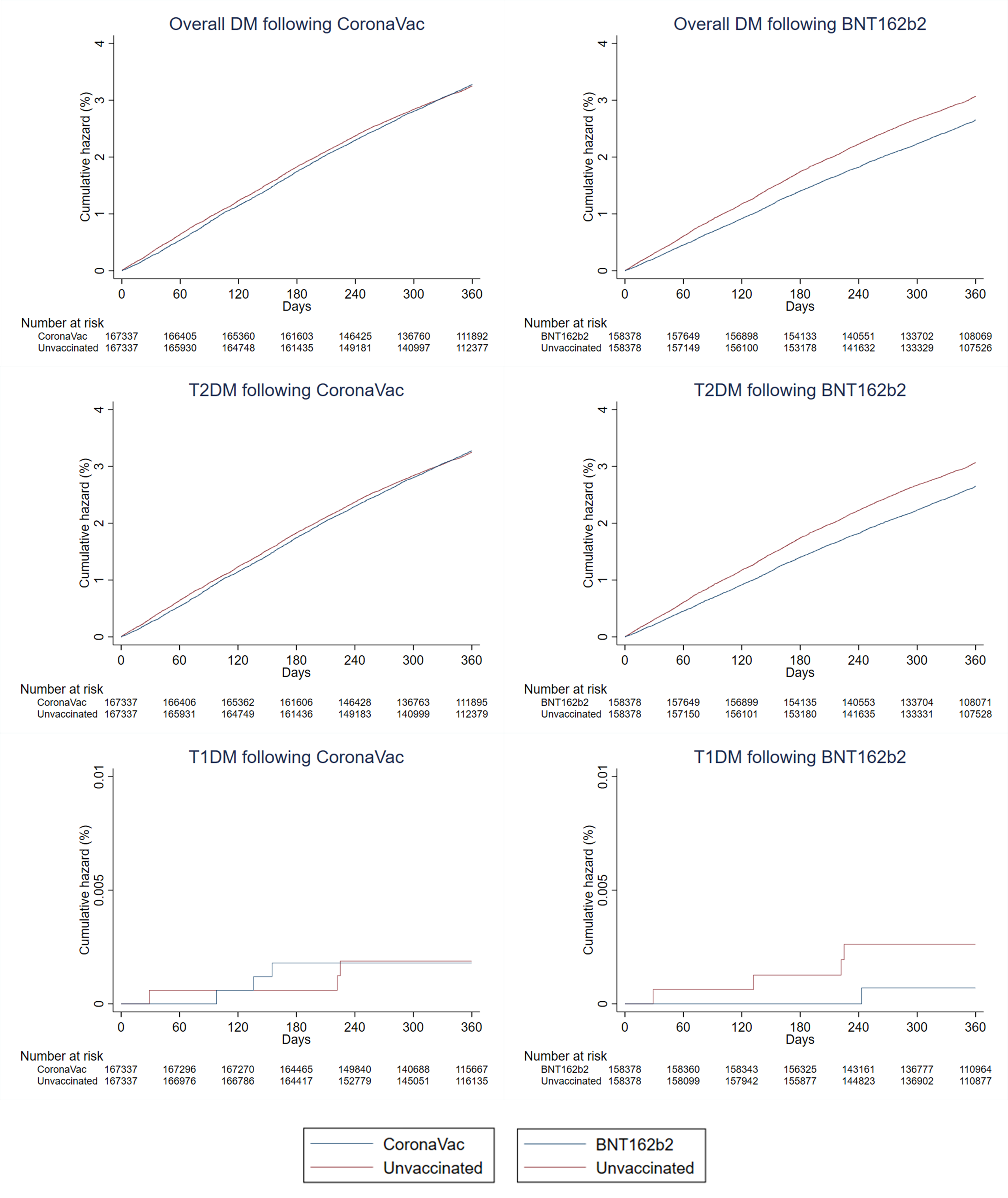

Supplement: S2 Fig — (TIF) [file pmed.1004274.s011.tif]

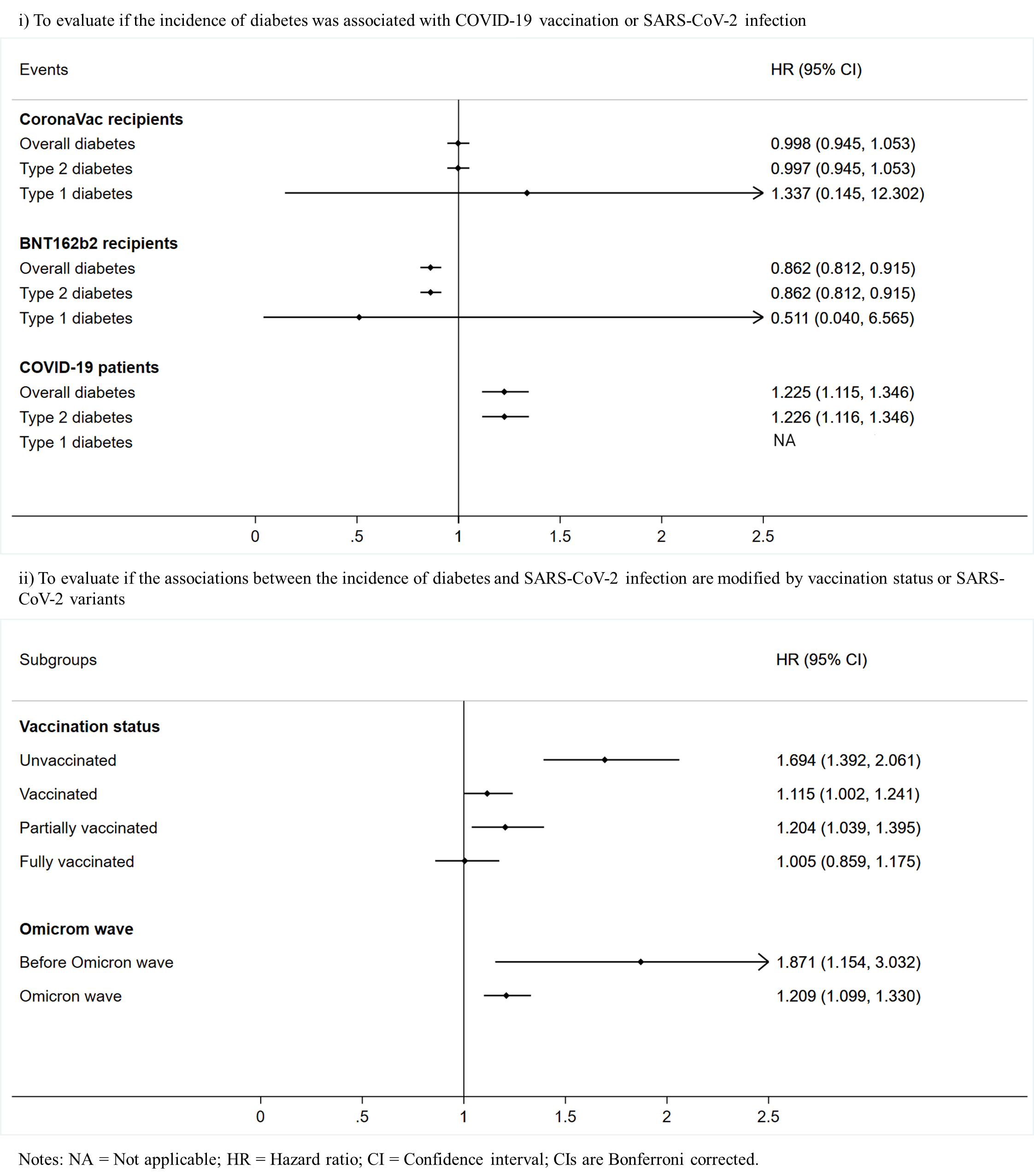

Supplement: S3 Fig — (TIF) [file pmed.1004274.s012.tif]

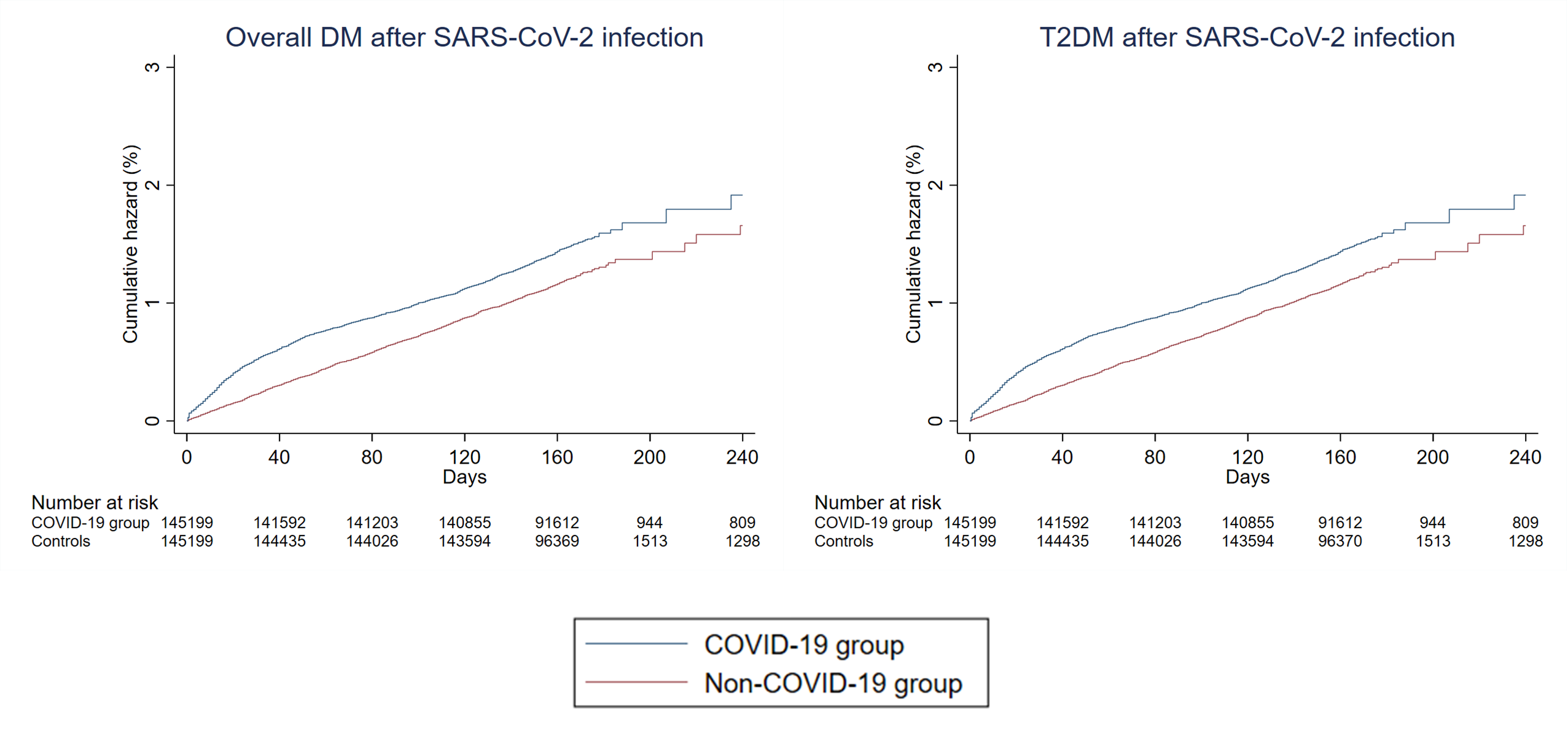

Supplement: S4 Fig — (TIF) [file pmed.1004274.s013.tif]
