# Supplementary material for: Incidence of diabetes following COVID-19 vaccination and SARS-CoV-2 infection in Hong Kong: A population-based cohort study
Source: PLoS Med. 2023 Jul 24;20(7):e1004274. doi: 10.1371/journal.pmed.1004274 (PMC10406181; doi:10.1371/journal.pmed.1004274)
Supplement: S1 Method — (DOCX) [file pmed.1004274.s014.docx]

S1 Method. COVID-19 vaccination policy in Hong Kong.

The mass COVID-19 vaccination program in Hong Kong was started on February 23, 2021 for CoronaVac (inactivated whole-virus vaccine) from Sinovac Biotech (Hong Kong) Limited and March 6, 2021 for BNT162b2 (monovalent mRNA vaccine) from BioNTech/Fosun Pharma in China (equivalent to the Pfizer-BioNTech vaccine outside China). At the very early stage, certain patient groups, like healthcare workers, were prioritised to receive vaccination. However, no guidelines recommend that certain individuals or patient groups receive a particular vaccine or different service delivery. The booking system, used by the Hong Kong Government, automatically schedules two doses of either vaccine for eligible citizens when they register for vaccination using a unique identity document number. Participants can select the preferred vaccine type and switch the vaccine type only after receiving the second dose. The government recommended receiving the third dose at least 90 days following the second dose for the general public not less than 18 years old.

As of September 2021, the vaccination uptake rate in Hong Kong was about 60.5% for the first dose (CoronaVac: 22.0%; BNT162b2: 38.5%) and 56.4% for the second dose of COVID-19 vaccine (CoronaVac: 20.5%; BNT162b2: 36.0%). People aged 20-59 years were more likely to receive COVID-19 vaccines, followed by people aged 60 or above. Generally, women are more likely to have received the COVID-19 vaccine than men[1].

**References**

1. COVID-19 Vaccination Programme. The Government of the Hong Kong Special Administrative Region. 2023. [accessed on 2023 April 02]. Available from: <https://www.covidvaccine.gov.hk/en/>.
