# Supplementary material for: Incidence of diabetes following COVID-19 vaccination and SARS-CoV-2 infection in Hong Kong: A population-based cohort study
Source: PLoS Med. 2023 Jul 24;20(7):e1004274. doi: 10.1371/journal.pmed.1004274 (PMC10406181; doi:10.1371/journal.pmed.1004274)
Supplement: S2 Method — (DOCX) [file pmed.1004274.s015.docx]

S2 Method. COVID-19 pandemic and key policies in Hong Kong.

The COVID-19 pandemic is a global outbreak of a respiratory illness caused by the novel coronavirus SARS-CoV-2. The outbreak was first identified in Wuhan, China in December 2019 and rapidly spread to other countries, eventually leading to a global pandemic.

The first case of COVID-19 in Hong Kong was identified on January 23, 2020, by a tourist from Wuhan. Despite the challenges posed by the pandemic, Hong Kong's response has been relatively successful in controlling the spread of the virus before 2022, with a lower number of confirmed cases.

While after 2022, the Omicron variant gradually became the predominant variant (so-called 'fifth wave'), leading to a significant increase in cases and hospitalizations in Hong Kong. The estimated cumulative incidence cases are up to 60.5% of Hong Kong's total population as of March 14, 2022[1]. The fifth wave of the COVID-19 pandemic prompted the government to implement more stringent measures, such as vaccine pass, mandatory mask-wearing and compulsory quarantine, to reduce the flow of people and contain transmission risk in the community. As of August 15, 2022 (the end of the follow-up period in our study), there have been a total of 833,574 confirmed cases of COVID-19 in Hong Kong, with 9,356 deaths[2].

Since September 26, 2022, as vaccination rates continued to increase and the number of cases began to decline, the Hong Kong Government lifted the compulsory quarantine requirement for inbound persons from overseas places or Taiwan[3].

**References**

1. Further update on estimation of Hong Kong’s infection figures in the fifth wave of COVID-19. D24H. 2022. [accessed on 2023 April 06]. Available from: <https://www.d24h.hk/news_event_article/further-update-on-estimation-of-hong-kongs-infection-figures-in-the-fifth-wave-of-covid-19/>.

2. Archive of Statistics on 5th Wave of COVID-19. The Government of the Hong Kong Special Administrative Region. 2023. [accessed on 2023 April 06]. Available from: <https://www.coronavirus.gov.hk/eng/5th-wave-statistics.html>.

3. Press Release: Government announces lifting of compulsory quarantine requirement on arrival at Hong Kong. The Government of the Hong Kong Special Administrative Region. 2022. [accessed on 2023 April 06]. Available from: <https://www.info.gov.hk/gia/general/202209/24/P2022092400048.htm?fontSize=1>.
