# Supplementary material for: Incidence of diabetes following COVID-19 vaccination and SARS-CoV-2 infection in Hong Kong: A population-based cohort study
Source: PLoS Med. 2023 Jul 24;20(7):e1004274. doi: 10.1371/journal.pmed.1004274 (PMC10406181; doi:10.1371/journal.pmed.1004274)
Supplement: S3 Method — (DOCX) [file pmed.1004274.s016.docx]

S3 Method. Propensity score matching.

Propensity score (PS) matching was considered a suitable method to reduce confounding arising from differences in baseline characteristics between vaccination recipients/COVID-19 patients and their respective controls.

In the analysis of risks following COVID-19 vaccination, logistic regression model was constructed to estimate the propensity score for each vaccination recipient and control based on the demographic and clinical variables included in previous propensity score weighted cohort study[1-5] and those which were likely to bias the number of incidents reported, including age (categorised into 16-44, 45-59, and ≥60 years), sex, prediabetes[6] (defined as baseline HbA1c ≥5.7% and <6.5%), Charlson comorbidity index (CCI), pre-existing comorbidities, medication use within the 90 days before the index date, and history of SARS-CoV-2 infection. The comorbidities, including the history of myocardial infarction, peripheral vascular disease, cerebrovascular disease, chronic obstructive pulmonary disease, dementia, paralysis, chronic renal failure, mild/moderate-severe liver disease, ulcers, rheumatoid arthritis or other inflammatory polyarthropathy, malignancy, mental disorders, and obesity, were defined by ICD-9-CM codes (S1 Table). The use of medications was defined by BNF codes (S2 Table). We used the nearest neighbour algorithm with a caliper of 0.05[7] to match CoronaVac/BNT162b2 recipients and unvaccinated people with a ratio of 1:1 separately.

In the analysis of risks following SARS-CoV-2 infection, logistic regression model was constructed to estimate the propensity score for each COVID-19 patient and control based on age, sex, prediabetes, CCI, pre-existing comorbidities, medication use within the 90 days before the index date, and vaccination status (unvaccinated, partially vaccinated and fully vaccinated). Fully vaccinated were defined as those with ≥2 doses of BNT162b2 or ≥3 doses of CoronaVac, following the definition in recent population-based studies[8, 9]. COVID-19 patients were 1:1 matched with non-COVID-19 people using the propensity score with a caliper width of 0.05.

A standardised mean difference (SMD) was used to assess the balance of baseline characteristics among the two groups, of which a value of <0.1 was considered acceptable[10].

**References**

1. Xiong X, Wong CKH, Au ICH, Lai FTT, Li X, Wan EYF, et al. Safety of Inactivated and mRNA COVID-19 Vaccination Among Patients Treated for Hypothyroidism: A Population-Based Cohort Study. Thyroid. 2022;32(5):505-14. doi: 10.1089/thy.2021.0684.

2. Lai FTT, Huang L, Chui CSL, Wan EYF, Li X, Wong CKH, et al. Multimorbidity and adverse events of special interest associated with Covid-19 vaccines in Hong Kong. Nat Commun. 2022;13(1):411. doi: 10.1038/s41467-022-28068-3.

3. Li X, Tong X, Yeung WWY, Kuan P, Yum SHH, Chui CSL, et al. Two-dose COVID-19 vaccination and possible arthritis flare among patients with rheumatoid arthritis in Hong Kong. Ann Rheum Dis. 2022;81(4):564-8. doi: 10.1136/annrheumdis-2021-221571.

4. Li X, Tong X, Wong ICK, Peng K, Chui CSL, Lai FTT, et al. Lack of inflammatory bowel disease flare-up following two-dose BNT162b2 vaccine: a population-based cohort study. Gut. 2022. doi: 10.1136/gutjnl-2021-326860.

5. Wong CKH, Xiong X, Lau KTK, Chui CSL, Lai FTT, Li X, et al. Impact of a delayed second dose of mRNA vaccine (BNT162b2) and inactivated SARS-CoV-2 vaccine (CoronaVac) on risks of all-cause mortality, emergency department visit, and unscheduled hospitalization. BMC Med. 2022;20(1):119. doi: 10.1186/s12916-022-02321-4.

6. Diagnosis: Understanding A1C. American Diabetes Association. 2022. [accessed on 2022 Nov 28]. Available from: <https://diabetes.org/diabetes/a1c/diagnosis>.

7. Ripollone JE, Huybrechts KF, Rothman KJ, Ferguson RE, Franklin JM. Implications of the Propensity Score Matching Paradox in Pharmacoepidemiology. Am J Epidemiol. 2018;187(9):1951-61. doi: 10.1093/aje/kwy078.

8. McMenamin ME, Nealon J, Lin Y, Wong JY, Cheung JK, Lau EHY, et al. Vaccine effectiveness of one, two, and three doses of BNT162b2 and CoronaVac against COVID-19 in Hong Kong: a population-based observational study. Lancet Infect Dis. 2022;22(10):1435-43. doi: 10.1016/S1473-3099(22)00345-0.

9. Yan VKC, Wan EYF, Ye X, Mok AHY, Lai FTT, Chui CSL, et al. Effectiveness of BNT162b2 and CoronaVac vaccinations against mortality and severe complications after SARS-CoV-2 Omicron BA.2 infection: a case-control study. Emerg Microbes Infect. 2022;11(1):2304-14. doi: 10.1080/22221751.2022.2114854.

10. Austin PC. Some methods of propensity-score matching had superior performance to others: results of an empirical investigation and Monte Carlo simulations. Biom J. 2009;51(1):171-84. doi: 10.1002/bimj.200810488.
