# Supplementary material for: Incidence of diabetes following COVID-19 vaccination and SARS-CoV-2 infection in Hong Kong: A population-based cohort study
Source: PLoS Med. 2023 Jul 24;20(7):e1004274. doi: 10.1371/journal.pmed.1004274 (PMC10406181; doi:10.1371/journal.pmed.1004274)
Supplement: S1 Protocol — (DOCX) [file pmed.1004274.s017.docx]

| 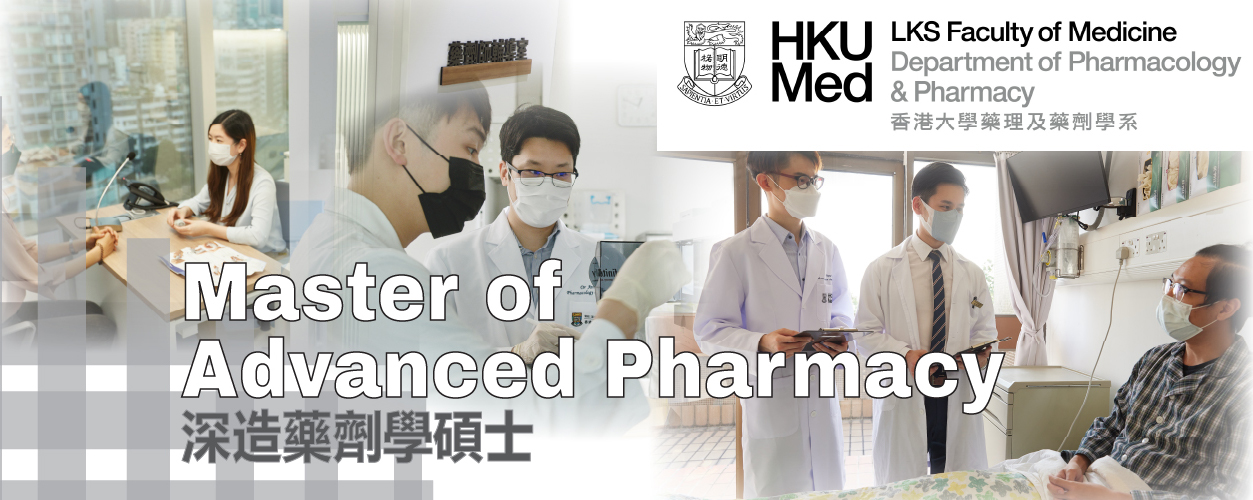 | Statistical Analysis Plan | | | | |
| --- | --- | --- | --- | --- | --- |
|  | Date | | 04 November 2022 | | |
|  | Authors | | 1. Xi Xiong 2. David Lui 3. Carlos Wong 4. Matthew Chung 5. Ivan Au | | |
|  |  | |  | | |
|  |  | |  | | |
|  |  | |  | | |
|  |  | |  | | |
| \| **Title of Proposed Research (with subtitle specifying the design, e.g., case-control)** \| \| --- \| \| Risk of incident diabetes mellitus associated with mRNA, inactivated COVID-19 vaccines and COVID-19 infection: a population-based cohort study  This Statistical Analysis Plan describes definitions and outcomes in this study, which will… (state objectives briefly, specifying the PICO, <100 words)  We will conduct a population-based study to evaluate the risks of incident diabetes mellitus associated with mRNA, inactivated COVID-19 vaccines and COVID-19 infection. \| | | | | | |
| title page | | | | | |
|  | | | | | |
| Principal investigator(s): | | Carlos Wong, David Lui, Xi Xiong | | | |
| Nominated main analyst(s): | | Xi Xiong | | | |
| Nominated second/third independent analyst(s): | | Matthew Chung/Ivan Au | | | |
| Publicly available on CSMPR or other websites? | | Yes  Date: | |  | No |

List Of Abbreviations

| **Abbreviation or special term** | **Explanation** |
| --- | --- |
| ACE2 | Angiotensin‐converting Enzyme 2 |
| CIs | Confidence Intervals |
| DH | Department of Health |
| DM | Diabetes Mellitus |
| EMRs | Electronic Medical Records |
| HA | Hospital Authority |
| HbA1c | Haemoglobin A1c |
| HRs | Hazard Ratios |
| ICD-9-CM | International Classification of Diseases, Ninth Revision, Coding Manual |
| ICPC | International Classification of Primary Care |
| PICO | Population, Intervention (or exposure), Comparator, and Outcome |
| RAT | Rapid Antigen Test |
| RT–PCR | Reverse Transcription Polymerase Chain Reaction |
| STROBE | STrengthening the Reporting of OBservational studies in Epidemiology |

| 1. | | BACKGROUND | | |  |
| --- | --- | --- | --- | --- | --- |
|  | | A number of studies have reported new-onset diabetes as being associated with the presence of COVID-19^1^. Binding of angiotensin‐converting enzyme 2 (ACE2) receptors, which are expressed in various metabolic tissues, including pancreatic beta cells, has been implicated^2^. These studies discussed point to a possible link between COVID-19 vaccine administration and resultant hyperglycemia and/or related complications. In view of the accumulating case reports and case series of hyperglycaemia following COVID-19 vaccination, there is an urgent need to clarify the association between COVID-19 vaccination and incident diabetes mellitus (DM) in a population-based study^3-5^.  However, case reports and series do not quantify the absolute risk of DM and inform if COVID-19 vaccination is associated with an increased risk of DM. Such information is essential to inform clinical practice for advising patients about COVID-19 vaccination, and the subsequent follow-up or surveillance. | | |  |
| 2. | | [OBJECTIVES](#_Toc351646345) | | |  |
|  | | This population-based study aims to evaluate the risks of incident DM following mRNA, inactivated COVID-19 vaccines and COVID-19 infection. | | |  |
| 3. | | [STUDY](#_Toc351646344) DESIGN | | |  |
| 3a | | Design: We will evaluate the risk of DM following COVID-19 vaccination and infection. | | | |
| 3b | | Data source: The study will be conducted using COVID-19-confirmed case records and COVID-19 vaccination records from the Department of Health (DH), and electronic medical records (EMRs) from the Hong Kong Hospital Authority (HA). Vaccination, infection and electronic health records are available for analysis up to 15th August 2022. **Setting of data:** inpatient and outpatient | | | |
| 3c | | Details of data handling: All eligible patients’ EMRs since 2018 will be extracted from the HA. The vaccination and infection records will be cross-linked with EMRs by hashed unique identifiers. | | | |
| 4. | | STUDY POPULATION | | |  |
|  | For evaluating risks following COVID-19 infection:COVID-19 patients who have a first positive result on the SARS-CoV-2 reverse transcription polymerase chain reaction (RT-PCR) test or rapid antigen test (RAT) from January 1, 2020, to March 31, 2022, and people who were never infected up to March 31, 2022 will be identified from HA system. | | |  |  |
| 4a | | | **Inclusion criteria**: i. Aged ≥18; | | |
| 4b | | | **Exclusion criteria**: i. Death on or before the index or pseudo-index date; ii. Without Haemoglobin A1c (HbA1c) measured before the index or pseudo-index date; iii. With incident diabetes mellitus before the index or pseudo-index date.  Incident DM is defined as (a) HbA1c levels >= 6.5% (48 mmol/mol) or; (b) prescription record of diabetes medications for more than 30 days (insulin: 6.1.1.x; oral hypoglycaemic drugs: 6.1.2.x) or; (c) ICD-9-CM 250.xx or; (d) ICPC: T89 and T90. | | |
|  | For evaluating risks following COVID-19 Vaccination:People who received at least one dose of COVID-19 vaccine from February 23, 2021, to September 30, 2021, and those who did not receive any COVID-19 vaccines up to September 30, 2021 will be identified from HA system. | | |  |  |
| 4a | | Inclusion criteria: i. Aged ≥18; | | | |
| 4b | | Exclusion criteria: i. Death on or before the index or pseudo-index date; ii. Without HbA1c measured before the index or pseudo-index date; iii. With incident diabetes mellitus before the index or pseudo-index date. | | | |
| 5 | STUDY OUTCOME | | |  |  |
| 5a | | Primary outcome: 1) DM; 2) type 1 DM (ICPC: T89 or; ICD-9: 250.x1, 250.x3); 3) type 2 DM (among patients with DM, they will be regarded as type 1 DM if they are not type 2 DM). | | | |
| 5b | | Secondary outcomes: 1) acute hyperglycaemia (ICD-9: 250.82–83, 250.20, 250.22–23); 2) diabetic ketoacidosis (ICD-9: 250.10, 250.12–13 and 250.30–33) | | | |
| 6 | EXPOSURE | | |  |  |
| 6a | | Primary exposure: COVID-19 vaccination | | | |
| 6b | | Secondary exposure: COVID-19 infection | | | |
| 7 | CONFOUNDERS/COVARIATES | | |  |  |
|  | List all confounders to be considered and how they are operationalized. e.g., medication use specific generic name and the period based on which they are defined. Can be a numbered list.  1. Age 2. Sex 3. Prediabetes (baseline HbA1c ≥ 5.7% and <6.5%) 4. Comorbidities 5. Use of medications 6. Previous SARS-CoV-2 infection (for vaccination cohort only) 7. Vaccination status (for infection cohort only) | | |  |  |
| 8 | EFFECT MODIFICATION/STRATIFICATION | | |  |  |
|  | Subgroup analyses will be conducted by age (<60 vs ≥60 years), sex, and prediabetes. In the infection cohort, we will also perform subgroup analyses by vaccination status, and stratify the participants into those who were infected before and those who were infected during the Omicron wave (since January 1, 2022). | | |  |  |
| 9 | ANALYSIS | | |  |  |
| 9a | | Main analysis: We will use propensity-score matching without replacement using a caliper width of 0.05. Hazard ratios (HRs) with 95% CIs of each outcome between vaccination recipients/COVID-19 patients and their respective matched controls will be estimated using Cox regression models. The number of acute hyperglycaemia and DKA following vaccination will be reported. | | | |
| 9b | | Sub-group analysis: analyses correspond to the stratified specified under Section 8. | | | |
| 9c | | Sensitivity analysis: Sensitivity analyses will be conducted. (i) We will compare the incidence of diabetes following two doses of either CoronaVac or BNT162b2 with their 1:1 matched unvaccinated control. (ii) People will be censored at the date of vaccination to remove potential modification effects by vaccination in the infection cohort. | | | |
| 10 | SAMPLE SIZE CONSIDERATION | | |  |  |
|  | The sample size is based on the number of patients without DM before vaccination and infection. | | |  |  |
| 11 | ANTICIPATED PITFALLS | | |  |  |
|  | Any possible ways the results could fail to achieve one or more of the study objectives.  1. Confounding bias 2. Misclassification bias | | |  |  |
| 12 | RELEVANT RESEARCH CHECKLIST | | |  |  |
|  | The STROBE checklist will be used to enhance the reporting of the study. | | |  |  |

| **REFERENCES:** |
| --- |

1. Khunti K, Del Prato S, Mathieu C, et al. COVID-19, Hyperglycemia, and New-Onset Diabetes. *Diabetes Care* 2021;44(12):2645-55. doi: 10.2337/dc21-1318 [published Online First: 2021/10/10]

2. Yang JK, Lin SS, Ji XJ, et al. Binding of SARS coronavirus to its receptor damages islets and causes acute diabetes. *Acta Diabetol* 2010;47(3):193-9. doi: 10.1007/s00592-009-0109-4 [published Online First: 2009/04/01]

3. Edwards AE, Vathenen R, Henson SM, et al. Acute hyperglycaemic crisis after vaccination against COVID-19: A case series. *Diabet Med* 2021;38(11):e14631. doi: 10.1111/dme.14631 [published Online First: 2021/06/30]

4. Lee HJ, Sajan A, Tomer Y. Hyperglycemic Emergencies Associated With COVID-19 Vaccination: A Case Series and Discussion. *J Endocr Soc* 2021;5(11):bvab141. doi: 10.1210/jendso/bvab141 [published Online First: 2021/10/05]

5. Tang X, He B, Liu Z, et al. Fulminant type 1 diabetes after COVID-19 vaccination. *Diabetes Metab* 2022;48(2):101324. doi: 10.1016/j.diabet.2022.101324 [published Online First: 2022/01/30]
