# Supplementary material for: Incidence of diabetes following COVID-19 vaccination and SARS-CoV-2 infection in Hong Kong: A population-based cohort study
Source: PLoS Med. 2023 Jul 24;20(7):e1004274. doi: 10.1371/journal.pmed.1004274 (PMC10406181; doi:10.1371/journal.pmed.1004274)
Supplement: Attachment — Submitted filename: Response to reviewers.docx [file pmed.1004274.s018.docx]

02 May 2023

Dr. Callam Davidson

Senior Editor

PLOS Medicine

**RE: PMEDICINE-D-23-00434R1- Incidence of diabetes following COVID-19 vaccination and SARS-CoV-2 infection: a population-based cohort study**

Dear Dr. Davidson,

On behalf of my co-authors, I would like to thank the reviewers for their valuable comments and suggestions. Responding to these, we have made the necessary adjustments in the revised manuscript, and hope that you will now find it suitable for publication. We provide here our point-to-point responses to the earlier version of this paper reviewed.

**Requests from the editors:

Abstract Methods and Findings:
* Please include the important dependent variables that are adjusted for in the analyses.**

Response E1.1: In the original analysis, we adjusted for previous SARS-CoV-2 infection when examining the association between vaccination status and incident diabetes, and vaccination status when examining the association between SARS-CoV-2 infection and incident diabetes. The sentence *‘The propensity score was generated by a logistic regression model conditional on age, sex, prediabetes, Charlson comorbidity index, pre-existing comorbidities and medication use. Since history of SARS-CoV-2 infection and vaccination status may influence incidence of diabetes, we also adjusted for previous SARS-CoV-2 infection when estimating the conditional probability of receiving vaccinations and vaccination status when estimating the conditional probability of contracting SARS-CoV-2 infection’* has been added to the Abstract Methods and Findings (line 62-68, page 3).

*** In the last sentence of the Abstract Methods and Findings section, please describe the main limitation(s) of the study's methodology.**

Response E1.2: The sentence *‘**The main limitations of our study included possible misclassification bias as type 1 diabetes was identified through diagnostic coding and possible residual confounders due to the observational nature of our stud*y’ has been added to the last sentence of the Abstract Methods and Findings (line 82-84, page 4).

**At this stage, we ask that you include a short, non-technical Author Summary of your research to make findings accessible to a wide audience that includes both scientists and non-scientists. The Author Summary should immediately follow the Abstract in your revised manuscript. This text is subject to editorial change and should be distinct from the scientific abstract. Please see our author guidelines for more information:** [**https://journals.plos.org/plosmedicine/s/revising-your-manuscript#loc-author-summary**](https://journals.plos.org/plosmedicine/s/revising-your-manuscript#loc-author-summary)
Response E1.3: An Author Summary is now added to pages 5-6 of the manuscript, immediately following the Abstract. Relevant section in text:

***Why was this study done?***

- *There have been an increasing number of cases of type 1 diabetes reported following COVID-19 vaccinations.*
- *The relationship between receiving COVID-19 vaccines and incident diabetes has not been examined in population-based studies.*
- *Several nationwide cohorts reported higher risks of incident diabetes following SARS-CoV-2 infection.*
- *The risk of incident diabetes following infection by SARS-CoV-2 Omicron variants may differ from that following infection by earlier variants. It is also uncertain how vaccination status may influence the risk.*

***What did the researchers do and find?***

- *This study included 167,337 CoronaVac, 158,378 BNT162b2 recipients, and 145,199 COVID-19 patients with their respective 1:1 matched control.*
- *Neither BNT162b2 nor CoronaVac was associated with increased risks of incident diabetes following COVID-19 vaccination.*
- *Regardless of predominant circulating variants, SARS-CoV-2 infection was associated with significantly higher risks of incident diabetes, particularly type 2 diabetes. However, these risks were lower with Omicron variants.*
- *Fully vaccinated COVID-19 survivors did not have an increased risk of incident diabetes.*

***What do these findings mean?***

- *There is still an increased risk of incident diabetes following SARS-CoV-2 infection even with the prevailing Omicron variants, although the risk is lower.*
- *Fully vaccinated individuals might be protected from the risk of incident diabetes following SARS-CoV-2 infection.*
- *As COVID-19 vaccination is not associated with increased risks of incident diabetes, our results encourage people to get fully vaccinated to protect themselves from severe complications of COVID-19 and the sequelae of long COVID, including the potential risk of incident diabetes.*

*This has been inserted on pages 5-6, lines 95-126.*

**Citations should be in square brackets, and preceding punctuation.**

Response E1.4: We ensured that all citations were in square brackets and proceeding punctuation.

**Please add the following statement, or similar, to the Methods: "This study is reported as per the Strengthening the Reporting of Observational Studies in Epidemiology (STROBE) guideline (S1 Checklist)."**

Response E1.5: The sentence *‘The study is reported as per the Strengthening the Reporting of Observational Studies in Epidemiology (STROBE) guideline (S1 Checklist)’* has been added to the Methods section (line 203-205, page 9-10).

**When completing the checklist, please use section headings and paragraph numbers, rather than page numbers.**

Response E1.6: The Reporting of Observational Studies in Epidemiology (STROBE) guideline (S1 Checklist) has been revised. We ensured that we used the section headings and paragraph numbers.

**Did your study have a prospective protocol or analysis plan? Please state this (either way) early in the Methods section.
a) If a prospective analysis plan (from your funding proposal, IRB or other ethics committee submission, study protocol, or other planning document written before analyzing the data) was used in designing the study, please include the relevant prospectively written document with your revised manuscript as a Supporting Information file to be published alongside your study, and cite it in the Methods section. A legend for this file should be included at the end of your manuscript.
b) If no such document exists, please make sure that the Methods section transparently describes when analyses were planned, and when/why any data-driven changes to analyses took place. 
c) In either case, changes in the analysis-- including those made in response to peer review comments-- should be identified as such in the Methods section of the paper, with rationale.**

Response E1.7: We had a prospective analysis plan which was uploaded the protocol document (date 04 November 2022 version 1.3) as Supporting Information (S1 Protocol)[1].

*Relevant text in the Data source of Methods section: “Furthermore, the data collection and analysis were based on a prospective protocol (S1 Protocol)[29].”* *(line 205-206, page 10)*

Reference:

1.	Proposed Protocol: Risk of incident diabetes mellitus associated with mRNA, inactivated COVID-19 vaccines and COVID-19 infection: a population-based cohort study COVID-19. Vaccines Adverse Events Response and Evaluation (CARE) Programme 2022 [accessed: April 21, 2023]. Available from: https://www.hkcare.hku.hk/protocol.

**Lines 243-245: Please remove the sentence relating to data availability as this information is already captured via the Submission Form questionnaire. Please note that a study author cannot be the primary contact for data requests.**

Response E1.8: We have removed the sentence *‘The datasets generated during and/or analysed during the current study are not publicly available due to no permission was given by data custodians but are available from the corresponding author upon reasonable request’* relating to data availability (line 355-357, page 16). We ensured that any study author would not be the primary contact for data requests.

**Line 310: Please avoid statements of primacy and include ‘to our knowledge’, or similar.**

Response E1.9: The statement *‘represent the first in the literature’* has been removed from the Discussion section (line 450-452, page 22).

**Lines 443-445: Please remove the lines relating to funding from the Acknowledgements (this already appears in the Financial Disclosure).**

Response E1.10: The sentence *‘F.T.T.L and I.C.K.W’s post were partly funded by D24H; hence this work was partly supported by AIR@InnoHK administered by Innovation and Technology Commission’* has been removed from the Acknowledgements section (line 607-609, page 28), and moved to Financial Disclosure section.

**Please ensure that any references to online-only sources include a date of accession.**

Response E1.11: We ensured that all online-only sources (Reference #1, #15, #17, #29, and #30) included a date of accession.

**Comments from the reviewers:

Reviewer #1:
This study evaluates the risk of a new diabetes diagnosis after Covid infection and Covid vaccination. The study draws on electronic health records data for a large population in Hong Kong. Through comparison with propensity score matched comparators, the study finds that Covid infection is associated with increased diabetes incidence, but Covid vaccination is not. The study suggests that diabetes risk is lower with the Omicron variant, and that vaccination status may influence risk of Covid-associated diabetes.

The findings with respect to Covid-19 infection and diabetes are confirmatory. However, the findings with respect to vaccination and vaccination status are more novel. The study appears to be generally well conducted. However, the clarity of the paper could be improved. Multiple different hypotheses are tested but the plan of analysis is not clear. Often, approaches with respect to different hypotheses are expressed in the same sentence.**

**Furthermore, the vaccination cohort was contaminated with Covid-19 infection, and the Covid-19 cohort by vaccination. The analytical approach may be pragmatic but these results are not fully transparent.**

Response R1.1: Thanks for your comments. In order to mitigate any potential effects of the contamination issue between these two cohorts, the following steps were taken. For the first cohort, we adjusted the history of SARS-CoV-2 infection in the propensity score model. After matching, only 0.3% of CoronVac recipients and 0.4% of BNT162b2 recipients have a history of SARS-CoV-2 infection (Table 1). The SMD of previous SARS-CoV-2 infection less than 0.1, representing the proportion of previous SARS-CoV-2 infection at baseline, were comparable between two groups. Meanwhile, people were censored at the date of SARS-CoV-2 infection to remove potential modification effects by infection in the main analysis when evaluating risks following COVID-19 vaccination. Hence, the estimated risks of incident diabetes following COVID-19 vaccination were less likely to be contaminated by the impact of SARS-CoV-2 infection.

For the second cohort, we adjusted vaccination status into the propensity score model. After matching, the SMD of vaccination status less than 0.1, representing vaccination status at baseline, were comparable between COVID-19 and non-COVID-19 people (Table 1). Additionally, people were censored at the date of vaccination to remove potential modification effects by vaccination in sensitivity analysis of evaluating risks following SARS-CoV-2 infection. Results were consistent with the main analysis in the sensitivity analyses after censoring at the date of vaccination (S5 Table). Hence, the estimated risks of incident diabetes following SARS-CoV-2 infection were less likely to be contaminated by the impact of COVID-19 vaccination.

**1. At line 134, please give more details on the provenance of the two vaccines. Also, in terms of context, please add in which jurisdictions the vaccines are in current use.**

Response R1.2: Thanks for your suggestions. The manufacturers of these two vaccines were Sinovac Biotech (Hong Kong) Limited for CoronaVac and Fosun Pharma in collaboration with the German drug manufacturer BioNTech for BNT162b2.

The sentence has been revised to *‘As of November 2022, the Hong Kong Government Vaccination Programme provides two main authorised COVID-19 vaccines: CoronaVac (inactivated whole-virus vaccine) from Sinovac Biotech (Hong Kong) Limited and BNT162b2 (monovalent mRNA vaccine) from BioNTech/Fosun Pharma in China (equivalent to the Pfizer-BioNTech vaccine outside China)’* in the Introduction section (line 176-180, page 8-9).

BNT162b2 is currently in use in many jurisdictions around the world, including North America (e.g. US, Canada, Mexico), South America (e.g. Brazil, Chile), Europe (e.g. UK, Switzerland), and Asia (e.g. Japan, Singapore). The jurisdictions where CoronaVac is currently in use are mostly located in Asia and Latin America, including China (where it was developed), Brazil, and Turkey.

*Relevant text in the Discussion section: “BNT162b2 is currently in use in many jurisdictions around the world, including North America (e.g. US, Canada, Mexico), South America (e.g. Brazil, Chile), Europe (e.g. UK, Switzerland), and Asia (e.g. Japan, Singapore). The jurisdictions where CoronaVac is currently in use are mostly located in Asia and Latin America, including China (where it was developed), Brazil, and Turkey.” (line 574-578, page 27)*

**2. Please also explain the vaccination policy. How were patients allocated to CoronaVac or BNT? Was the policy for two doses of the same vaccine or one dose of each? (as noted at line 221).**

Response R1.3: Thanks for your suggestions. The details of the vaccination policy have been described in the S1 Method and the relevant sentence has been added to the Methods section.

*Relevant text in the S1 Method: “The mass COVID-19 vaccination program in Hong Kong was started on February 23, 2021 for CoronaVac (inactivated whole-virus vaccine) from Sinovac Biotech (Hong Kong) Limited and March 6, 2021 for BNT162b2 (monovalent mRNA vaccine) from BioNTech/Fosun Pharma in China (equivalent to the Pfizer-BioNTech vaccine outside China). At the very early stage, certain patient groups, like healthcare workers, were prioritised to receive vaccination. However, no guidelines recommend that certain individuals or patient groups receive a particular vaccine or different service delivery. The booking system, used by the Hong Kong Government, automatically schedules two doses of either vaccine for eligible citizens when they register for vaccination using a unique identity document number. Participants can select the preferred vaccine type and switch the vaccine type only after receiving the second dose. The government recommended receiving the third dose at least 90 days following the second dose for the general public not less than 18 years old.”*

*Relevant text in the Methods section: “The COVID-19 vaccination policy and vaccine uptake rate in Hong Kong have been elaborated in S1 Method.” (line 223-224, page 10)*

**3. For context, please mention the vacccine uptake rates and associations with sociodemographic variables.**

Response R1.4: Thanks for your suggestions. The vaccine uptake rates have been reported in the S1 Method and the relevant sentence has been added to the Methods section.

*Relevant text in the S1 Method: “As of September 2021, the vaccination uptake rate in Hong Kong was about 60.5% for the first dose (CoronaVac: 22.0%; BNT162b2: 38.5%) and 56.4% for the second dose of COVID-19 vaccine (CoronaVac: 20.4%; BNT162b2: 36.0%). People aged 20-59 years were more likely to receive COVID-19 vaccines, followed by people aged 60 or above. Generally, women are more likely to have received the COVID-19 vaccine than men[1].”*

*Relevant text in the Methods section: “The COVID-19 vaccination policy and vaccine uptake rate in Hong Kong have been elaborated in S1 Method.” (line 223-224, page 10)*

Reference:
1.	COVID-19 Vaccination Programme The Government of the Hong Kong Special Administrative Region; 2023 [accessed: April 02, 2023]. Available from: https://www.covidvaccine.gov.hk/en/.

**4. At line 168, those who did not receive any vaccines were controls presumably and not part of the vaccine cohort, it is not made clear.**

Response R1.5: Sorry for being unclear. Yes, those who did not receive any vaccines were defined as the controls in the analysis of diabetes risks following COVID-19 vaccination. We have changed the term *‘The vaccination cohort’* to ‘*The first cohort for evaluating risks following COVID-19 vaccination (the first cohort)’*, and *‘The infection cohort’* to *‘The second cohort for evaluating risks following SARS-CoV-2 infection (the second cohort)’* in the Methods section (line 218-219, page 10; line 236-237, page 11).

**5. The statistical analysis section could be clearer and and analysis plan should be provided as supplementary material. There are several hypotheses: that vaccination is associated with diabetes; that the two vaccines differ; that Covid-19 is associated with diabetes; that the risk varies for different variants; that the effect of Covid 19 is modified by vaccination etc. It should be explained how each hypothesis is tested. How will type 1 error rate be controlled?**

Response R1.6: Thanks. The details of the propensity score matching have been moved to S3 Method. In our study, we tested the following four hypotheses: (i) the incidence of diabetes was associated with COVID-19 vaccination, (ii) the incidence of diabetes was associated with SARS-CoV-2 infection, (iii) the association between the incidence of diabetes and SARS-CoV-2 infection varies for SARS-CoV-2 variants, and (iv) the association between the incidence of diabetes and SARS-CoV-2 infection modified by vaccination status. The statistical analysis plan for different hypothesis testing has been revised.

*Relevant text in the Statistical analysis of the Methods section:* *“The association between incident diabetes and COVID-19 vaccination was estimated through Cox proportional hazards regression. Analyses were repeated to estimate the association between incident diabetes and SARS-CoV-2 infection.* *Crude incidence rates per 100 persons and cumulative incidence rates per 100,000 person-days were reported for vaccination recipients, COVID-19 patients and their respective control groups. Then, we evaluated the hazard ratios (HRs) and corresponding 95% confidence intervals (CIs) of incident diabetes.*

*To evaluate if the association between SARS-CoV-2 infection and incident diabetes was modified by vaccination or predominant variants, we also performed subgroup analyses by vaccination status and stratified the participants into those who were infected before and those who were infected during the Omicron wave (since January 1, 2022). HRs with 95%CIs were calculated for each subgroup analysis. P values for interaction terms were calculated for each stratifying variable.”* *(line 275-303, page 12-14; line 311-316, page 14)*

For controlling the type 1 error rate, the Bonferroni correction was applied to account for multiple testing. We set the α level (probability of type I error) to 0.05/15 (3 outcomes × 3 exposures + 1 outcome × 4 vaccination status + 1 outcome × 2 SARS-CoV-2 variants = 15 tests) = 0.00333 and calculated Bonferroni corrected CIs for HRs[1]. The results with Bonferroni correction were consistent with those without Bonferroni correction (S3 Fig). The relevant sentences have been added to the Methods and Results sections.

*Relevant text in the Statistical analysis of the Methods section: “We performed four post-hoc sensitivity analyses. First, the Bonferroni correction was applied to account for multiple hypotheses testing. We set the α level (probability of type I error) to 0.05/15 (number of analyses) = 0.00333 and calculated Bonferroni corrected CIs for HRs[35].” (line 324-326, page 14)*

*Relevant text in the Results section: “In the post-hoc analysis, the results with Bonferroni correction were consistent with those without Bonferroni correction (S3 Fig).”* *(line 441-442, page 22)*

Reference:
1.	Abdi H. Bonferroni and Šidák corrections for multiple comparisons. Encyclopedia of measurement and statistics. 2007;3:103-7.

**It is unclear, for example, how propensity score matching was used. Controls, it seems, were matched on age and sex only? (lines 259 to 263 refer to 'controls' and 'matched controls'; lines 274-5 refer to 'matched controls' and 'matched unvaccinated people').**

Response R1.7: Sorry for being unclear. The details of the propensity score matching have been described in S3 Method.

The matching based on age and sex was conducted to assign pseudo-index dates for unvaccinated and non-COVID-19 people, following the method described in previous studies[1-4]. After assigning the pseudo-index dates, we defined the baseline characteristics for unvaccinated and non-COVID-19 people. The follow-up period started from a pseudo-index date for unvaccinated and non-COVID-19 people. The details of the method to assign pseudo-index dates have been elaborated in the Methods section.

*Relevant text in the Study design and population of the Methods section: “To assign pseudo-index dates for unvaccinated people, we matched them with vaccine recipients based on sex and age. To ensure each unvaccinated person could match with a vaccine recipient, the maximum ratio was used. Afterwards, the index date of vaccine recipients was assigned to corresponding unvaccinated people.*

*To assign pseudo-index dates for people without infection, we matched them with COVID-19 patients based on sex and age. To ensure each non-COVID-19 person could match with a COVID-19 patient, the maximum ratio was used. The index date of COVID-19 patient was then assigned to corresponding non-COVID-19 people.”* *(line 227-230, page 10-11; line 244-248, page 11)*

After defining the baseline characteristics, propensity score matching was performed to reduce confounding arising from differences in baseline characteristics between vaccination recipients/COVID-19 patients and their respective controls. The propensity score was generated by a logistic regression model conditional on age (categorised into 16-44, 45-59, and ≥60 years), sex, prediabetes, Charlson comorbidity index (CCI), pre-existing comorbidities and medication use. Since history of SARS-CoV-2 infection and vaccination status may influence incident diabetes, we also adjusted for previous SARS-CoV-2 infection when estimating the conditional probability of receiving vaccinations and vaccination status when estimating the conditional probability of contracting SARS-CoV-2 infection. The relevant sentences have been added to the Methods. The details of propensity score matching have been described in the S3 Method.

*Relevant text in the Statistical analysis of the Methods section: “We conducted a propensity-score matched cohort study, matching participants and controls at a 1:1 ratio using the propensity score (see S3 Method for details).”* *(line 272-273, page 12)*

*Relevant text in the S3 Method: “Propensity score (PS) matching was considered a suitable method to reduce confounding arising from differences in baseline characteristics between vaccination recipients/COVID-19 patients and their respective controls.*

*In the analysis of risks following COVID-19 vaccination, logistic regression model was constructed to estimate the propensity score for each vaccination recipient and control based on the demographic and clinical variables included in previous propensity score weighted cohort study[5-9] and those which were likely to bias the number of incidents reported, including age (categorised into 16-44, 45-59, and ≥60 years), sex, prediabetes[10] (defined as baseline HbA1c ≥5.7% and <6.5%), Charlson comorbidity index (CCI), pre-existing comorbidities, medication use within the 90 days before the index date, and history of SARS-CoV-2 infection. The comorbidities, including the history of myocardial infarction, peripheral vascular disease, cerebrovascular disease, chronic obstructive pulmonary disease, dementia, paralysis, chronic renal failure, mild/moderate-severe liver disease, ulcers, rheumatoid arthritis or other inflammatory polyarthropathy, malignancy, mental disorders, and obesity, were defined by ICD-9-CM codes (S1 Table). The use of medications was defined by BNF codes (S2 Table). We used the nearest neighbour algorithm with a caliper of 0.05[11] to match CoronaVac/BNT162b2 recipients and unvaccinated people with a ratio of 1:1 separately.*

*In the analysis of risks following SARS-CoV-2 infection, logistic regression model was constructed to estimate the propensity score for each COVID-19 patient and control based on age, sex, prediabetes, CCI, pre-existing comorbidities, medication use within the 90 days before the index date, and vaccination status (unvaccinated, partially vaccinated and fully vaccinated). Fully vaccinated were defined as those with ≥2 doses of BNT162b2 or ≥3 doses of CoronaVac, following the definition in recent population-based studies[12, 13]. COVID-19 patients were 1:1 matched with non-COVID-19 people using the propensity score with a caliper width of 0.05.*

*A standardised mean difference (SMD) was used to assess the balance of baseline characteristics among the two groups, of which a value of <0.1 was considered acceptable[14].”*

References:

1.	Li X, Tong X, Yeung WWY, Kuan P, Yum SHH, Chui CSL, et al. Two-dose COVID-19 vaccination and possible arthritis flare among patients with rheumatoid arthritis in Hong Kong. Ann Rheum Dis. 2022;81(4):564-8. Epub 2021/10/24. doi: 10.1136/annrheumdis-2021-221571. PubMed PMID: 34686479.

2.	Lai FTT, Huang L, Chui CSL, Wan EYF, Li X, Wong CKH, et al. Multimorbidity and adverse events of special interest associated with Covid-19 vaccines in Hong Kong. Nat Commun. 2022;13(1):411. Epub 2022/01/22. doi: 10.1038/s41467-022-28068-3. PubMed PMID: 35058463.

3.	Lai FTT, Chua GT, Chan EWW, Huang L, Kwan MYW, Ma T, et al. Adverse events of special interest following the use of BNT162b2 in adolescents: a population-based retrospective cohort study. Emerg Microbes Infect. 2022;11(1):885-93. Epub 2022/03/08. doi: 10.1080/22221751.2022.2050952. PubMed PMID: 35254219.

4.	Kang W, Shami JJP, Yan VKC, Ye X, Blais JE, Li X, et al. Safety of two-dose COVID-19 vaccination (BNT162b2 and CoronaVac) in adults with cancer: a territory-wide cohort study. J Hematol Oncol. 2022;15(1):66. Epub 2022/05/20. doi: 10.1186/s13045-022-01265-9. PubMed PMID: 35590336.

**6.** **Choice of the Cox model may not be optimal since it is likely that any increase in risk is transient after vaccination or infection events, whereas the hazard ratio represents an average over the time of study.**

Response R1.8: Thanks for your comments. To address this concern, we have added a sensitivity analysis that evaluated the association between COVID vaccination/infection and incident diabetes by using Poisson regression. The incidence rate ratios (IRRs) and corresponding 95% confidence intervals (CIs) of incident diabetes were estimated to compare the risks between COVID vaccination/infection and their respective matched controls. The results from the Poisson regression were consistent with the result of the main analysis (S7 Table). The relevant sentences have been added to Methods and Results sections.

*Relevant text in the Statistical analysis of the Methods section: “Third, we evaluated the associations between COVID vaccination/infection and incident diabetes using Poisson regression after propensity score matching. The incidence rate ratios (IRRs) and corresponding 95% CIs of incident diabetes were estimated to compare the risks between COVID vaccination/infection and their respective matched controls.” (line 339-342, page 15)*

*Relevant text in the Results section: “The results of the analysis, which was performed using Poisson regression and IPTW-weighted Cox regression, were in line with the findings of the main analysis (S7-8 Table).”* *(line 444-446, page 22)*

**7. How will type 1 error be controlled, and rather than referring to P<0.05 as significant, please use the ASA guidelines on P values as quantifying the strength of evidence.**
[**https://www.tandfonline.com/doi/full/10.1080/00031305.2016.1154108**](https://www.tandfonline.com/doi/full/10.1080/00031305.2016.1154108)

Response R1.9: Thanks for your suggestions. For controlling the type 1 error rate, the Bonferroni correction was applied to account for multiple hypotheses testing. We set the α level (probability of type I error) to 0.05/15 (number of analyses) = 0.00333 and calculated Bonferroni corrected CIs for HRs[1]. The results with Bonferroni correction were consistent with those without Bonferroni correction (S3 Fig). The relevant sentences have been added to the Methods and Results sections.

*Relevant text in the Statistical analysis of the Methods section: “We performed four post-hoc sensitivity analyses. First, the Bonferroni correction was applied to account for multiple hypotheses testing. We set the α level (probability of type I error) to 0.05/15 (number of analyses) = 0.00333 and calculated Bonferroni corrected CIs for HRs[35].” (line 324-326, page 14)*

*Relevant text in the Results section: “In the post-hoc analysis, the results with Bonferroni correction were consistent with those without Bonferroni correction (S3 Fig).”* *(line 441-442, page 22)*

Following the recommendation of the American Statistical Association[2], we presented 95% of two-sided CIs and interpreted the results based on point estimates with their respective CIs. With reporting the Bonferroni-corrected CIs in the S3 Fig, the p-values < 0.05 would not be interpreted as statistically significant. The relevant sentences have been added to the Methods section.

*Relevant text in the Statistical analysis of the Methods section: “Following the recommendation of the American Statistical Association[40], we presented 95% of two-sided CIs and interpreted the results based on point estimates with their respective CIs.”* *(line 348-350, page 15)*

References:

1.	Abdi H. Bonferroni and Šidák corrections for multiple comparisons. Encyclopedia of measurement and statistics. 2007;3:103-7.

2.	Wasserstein RL, Lazar NA. The ASA Statement on p-Values: Context, Process, and Purpose. The American Statistician. 2016;70(2):129-33. doi: 10.1080/00031305.2016.1154108.

**8. Line 268, state how many cases of diabetes were identified.**

Response R1.10: Thanks for your suggestions. In the first cohort, we identified 5,760 and 4,411 diabetes cases after receiving CoronaVac and BNT162b2 vaccines, respectively. In the second cohort, we identified 2,109 diabetes cases following SARS-CoV-2 infection. The relevant sentences have been added to Results section.

*Relevant text in the Results section: “In the first cohort, we identified 5,760 and 4,411 diabetes cases after receiving CoronaVac and BNT162b2 vaccines, respectively.*

*In the second cohort, we identified 2,109 cases of diabetes following SARS-CoV-2 infection.” (line 385-386, page 19; line 416-417, page 21)*

**9. Looking at Table 1, it may appear surprising that half of all participants had pre-diabetes as noted in the discussion.**

Response R1.11: Thanks for your comments. A previous study has reported a prevalence of prediabetes of up to 40% among the Chinese population[1]. In our study, the proportion of participants with prediabetes before propensity score matching ranged from 45% to 53% (S3 Table). It is possible that the slightly higher prevalence of prediabetes observed in the present study is linked to the study’s requirement for valid HbA1c values as a criterion for excluding individuals with pre-existing diabetes. Individuals who have undergone HbA1c testing might have a higher risk of cardiometabolic complications and have different lifestyles, like being overweight or lack of physical activity which may contribute to the development of prediabetes. We acknowledged it as one of the limitations in the Discussion section.

*Relevant text in the Discussion section:* *“Third, the proportion of prediabetes in the current study cohort was around 50%, where the prevalence of prediabetes has been reported to be up to 40% among Chinese[1]. The slightly higher proportion of prediabetes individuals in the current study might be due to the requirement of valid HbA1c values before cohort entry for exclusion of individuals with pre-existing diabetes, where HbA1c might have been checked among individuals with relatively higher cardiometabolic risks.”* *(line 580-585, page 27)*

Reference:

1. Wang L, Gao P, Zhang M, Huang Z, Zhang D, Deng Q, et al. Prevalence and Ethnic Pattern of Diabetes and Prediabetes in China in 2013. JAMA. 2017;317(24):2515-23. Epub 2017/06/28. Doi: 10.1001/jama.2017.7596. PubMed PMID: 28655017.

**10. Also in Table 1, it may not be clear why 0% of the vaccination cohort are fully vaccinated but more than 40% of the Covid-19 patients are fully vaccinated. (add foot note)**

Response R1.12: Thanks for your comments. In table 1, we showed the characteristic of the study population at the baseline. For the first cohort, we included people who received at least one dose, and the index date was defined as the date of the first dose. Hence, the vaccination recipients only received one dose at the baseline and might not be categorized as fully vaccinated.

**11. Table 2, the ‘Rate’ and ‘Estimate’ are ambiguously labelled and the ‘rate’ could perhaps be omitted.**

Response R1.13: Sorry for the unclear description. We have changed the ‘Rate’ to ‘Cumulative incidence rate’, and ‘Estimate’ to ‘Crude incidence rate’ in the Table 2, S4 Table, S5 Table, and S7-8 Table.

**12. Table 3 may be better presented as a Forest plot. Type 1 diabetes data is uninformative.**

Response R1.14: Thanks for your suggestions. The Table 3 has been present as a forest plot for all the subgroup analyses (Fig 2). We followed the suggestions from both Reviewer 1 and Reviewer 2, and only reported the result of the overall diabetes.

**Reviewer #2: Alex McConnachie, Statistical Review

The paper by Xi Xiong and colleagues examines the incidence of diabetes following COVID-19 vaccination or infection, using national public health datasets from Hong Kong. This review considers the use of statistics in the paper.

In general, the statistical methods look good.

The exposed and unexposed cohorts are clearly defined. Propensity score matching is used to control for confounding due to measurable differences between the exposed and unexposed cohorts. The matching procedure looks good, though I have read some criticisms of the nearest neighbour within caliper method; did the authors consider any alternative approaches to using the propensity scores (as a sensitivity analysis, perhaps), such as inverse probability weighting, or an alternative matching procedure?**

Response R2.1: Thanks for your suggestions. We have added a sensitivity analysis that Cox regression models weighted by the inverse probability of treatment weighting (IPTW) were fitted to estimate the risks of incident diabetes following COVID-19 vaccination and infection, expressed in terms of hazard ratio (HR) and 95% confidence interval (CI). Inverse probability of treatment weighting (IPTW) was performed using the propensity score. Weight truncation at the 1^st^ and 99^th^ percentile of the observed propensity score weighting distribution was applied to address extreme weights. The results of the analysis, which was performed using IPTW-weighted Cox regression, were in line with the findings of the main analysis (S8 Table). The relevant sentences have been added to the Methods and Results sections.

*Relevant text in the Statistical analysis of the Methods section: “Fourth, we used the propensity score to perform inverse probability of treatment weighting (IPTW) and truncated the weight at the 1^st^ and 99^th^ percentile of the observed PS weighting distribution to address extreme weights. Cox regression models weighted by the IPTW were fitted to estimate the risks of incident diabetes following COVID-19 vaccination and infection.” (line 342-346, page 15)*

*Relevant text in the Results section: “The results of the analysis, which was performed using Poisson regression and IPTW-weighted Cox regression, were in line with the findings of the main analysis (S7-8 Table).”* *(line 444-446, page 22)*

**Cox models are used to estimate associations. It is not clear whether and how the analysis takes account of the pair-matched structure in the data. Also,** **were the proportional hazards assumptions checked for these models? These points could be clarified.**

Response R2.2: Thank you for your comments. We would like to clarify these points. To account of the pair-matched structure in the data, we used a cluster-robust sandwich variance–covariance estimator in all Cox regression models in the original analysis. The relevant sentence has been added to the Methods section.

*Relevant text in the Statistical analysis of the Methods section: “To account of the pair-matched structure in the data, we used a cluster-robust sandwich variance–covariance estimator in all Cox regression models.”* *(line 303-305, page 14)*

We tested the proportional hazards (PH) assumption in the Cox regression for main analyses, where the PH assumption is violated. Therefore, we performed the restricted mean survival time (RMST) analysis which is suggested as a supplement to the Cox PH model analysis without relying on the PH assumption[1-2]. Compared with Cox PH model analysis, RMST offered more interpretable metrics which have been recently applied in a variety of domains, including evaluating the treatment effect in people with type 2 diabetes[3-4]. The RSMT difference and restricted mean time lost (RMTL) ratio for each outcome were calculated. The RMST difference refers to the average event-free survival time difference over a restricted time horizon. RMTL represents the region above the Kaplan-Meier survival curve and indicates the average event-free survival time lost up to a restricted time horizon. The hazard ratio is proposed to be approximated using the RMTL ratio between treatment groups without imposing the PH assumption[2]. In our analysis, the time horizon for a given outcome was determined as the minimum of the largest followed-up time (99%) of vaccine recipients/COVID-19 patients and their matched controls.

The results from the RMST analysis are shown as below. The RMST difference was consistent with the direction of the HRs from Cox regressions, and numerical values were close between HRs and RMTL ratios. The relevant sentences have been added to Methods and Results sections. The results have been added to S6 Table.

Table 1. The restricted mean survival time (RMST) difference and the restricted mean time lost (RMTL) ratio for outcomes.

| Events | RMST difference* (Days) | | 95% CI | | P-value | RMTL ratio† | | 95% CI | | | P-value | | Time horizon (Days) |
| --- | --- | --- | --- | --- | --- | --- | --- | --- | --- | --- | --- | --- | --- |
| **CoronaVac recipients** | |  | |  |  | |  | |  |  | |  |  |
| **Overall diabetes** | 0.239 | | (-0.236, 0.714) | | 0.323 | 0.982 | | (0.946, 1.018) | | | 0.323 | | 532 |
| **Type 2 diabetes** | 0.242 | | (-0.233, 0.716) | | 0.318 | 0.981 | | (0.946, 1.018) | | | 0.318 | | 532 |
| **Type 1 diabetes** | -0.002 | | (-0.014, 0.01) | | 0.769 | 1.256 | | (0.272, 5.788) | | | 0.770 | | 532 |
| **BNT162b2 recipients** | |  | |  |  | |  | |  |  | |  |  |
| **Overall diabetes** | 1.827 | | (1.383, 2.27) | | <0.001 | 0.846 | | (0.812, 0.881) | | | <0.001 | | 521 |
| **Type 2 diabetes** | 1.822 | | (1.379, 2.265) | | <0.001 | 0.846 | | (0.812, 0.881) | | | <0.001 | | 521 |
| **Type 1 diabetes** | 0.005 | | (-0.006, 0.017) | | 0.364 | 0.454 | | (0.082, 2.514) | | | 0.366 | | 522 |
| **COVID-19 patients** | |  | |  |  | |  | |  |  | |  |  |
| **Overall diabetes** | -0.501 | | (-0.607, -0.395) | | <0.001 | 1.385 | | (1.293, 1.483) | | | <0.001 | | 189 |
| **Type 2 diabetes** | -0.501 | | (-0.608, -0.395) | | <0.001 | 1.385 | | (1.293, 1.484) | | | <0.001 | | 189 |
| **Type 1 diabetes** | NA | | NA | | NA | NA | | NA | | | NA | | 189 |

Notes: RMST = Restricted mean survival time; RMTL = Restricted mean time lost; CI = Confidence interval; NA = Not applicable

* RMST difference > 0 (or < 0) indicates vaccine recipients or COVID-19 patients have a longer (or shorter) average of event-free survival time than the matched controls during a specified time horizon.

† RMTL ratio < 1 (or > 1) indicates the vaccine recipients or COVID-19 patients lose less (or more) event-free survival time than the matched controls during the specified time horizon on average.

*Relevant text in the Statistical analysis of the Methods section: “Second, we performed the restricted mean survival time (RMST) analysis, which is suggested as a supplement to the Cox PH model analysis without relying on the PH assumption[36, 37]. Compared with the Cox PH model analysis, RMST offered more interpretable metrics which have been recently applied in a variety of domains, including evaluating the treatment effect in people with type 2 diabetes[38, 39]. The RSMT difference and restricted mean time lost (RMTL) ratio for each outcome were calculated. The RMST difference refers to the average event-free survival time difference over a restricted time horizon. RMTL represents the region above the Kaplan-Meier survival curve and indicates the average event-free survival time lost up to a restricted time horizon. The hazard ratio is proposed to be approximated using the RMTL ratio between treatment groups without imposing the PH assumption[37]. In our analysis, the time horizon for a given outcome was determined as the minimum of the largest followed-up time (99%) of vaccine recipients/COVID-19 patients and their matched controls.” (line 327-338, page 14-15)*

*Relevant text in the Results section: “The results from the RMST analysis are shown in S6 Table. The RMST difference was consistent with the direction of the HRs from Cox regressions, and numerical values were close between HRs and RMTL ratios.”* *(line 444-446, page 22)*

References:

1.	Kloecker DE, Davies MJ, Khunti K, Zaccardi F. Uses and Limitations of the Restricted Mean Survival Time: Illustrative Examples From Cardiovascular Outcomes and Mortality Trials in Type 2 Diabetes. Ann Intern Med. 2020;172(8):541-52. Epub 2020/03/24. doi: 10.7326/m19-3286. PubMed PMID: 32203984.

2.	Uno H, Claggett B, Tian L, Fu H, Huang B, Kim DH, et al. Adding a new analytical procedure with clinical interpretation in the tool box of survival analysis. Ann Oncol. 2018;29(5):1092-4. Epub 2018/04/05. doi: 10.1093/annonc/mdy109. PubMed PMID: 29617717; PubMed Central PMCID: PMCPMC5961386.

3.	Uno H, Claggett B, Tian L, Inoue E, Gallo P, Miyata T, et al. Moving beyond the hazard ratio in quantifying the between-group difference in survival analysis. J Clin Oncol. 2014;32(22):2380-5. Epub 2014/07/02. doi: 10.1200/jco.2014.55.2208. PubMed PMID: 24982461; PubMed Central PMCID: PMCPMC4105489 are found at the end of this article.

4.	Ferrannini E, Rosenstock J. Clinical Translation of Cardiovascular Outcome Trials in Type 2 Diabetes: Is There More or Is There Less Than Meets the Eye? Diabetes Care. 2021;44(3):641-6. Epub 2021/02/21. doi: 10.2337/dc20-0913. PubMed PMID: 33608325; PubMed Central PMCID: PMCPMC7896267.

**Subgroup analyses are performed, and interaction p-values are reported (in the tables) for these, which is good. When reported in the text, only the lack of associations between infection and diabetes in younger people, and in those fully vaccinated, are reported - it is not made clear that these represent significant interactions, and that the associations are stronger in older, and particularly unvaccinated individuals. Also, the interaction with prediabetes is not mentioned - it is only stated that the results are consistent with the overall results, despite an apparently stronger association in those without diabetes. Admittedly, this may be something to do with the inclusion criteria that required an HbA1c measurement.**

Response R2.3: Thanks for your suggestions. The sentence ‘*The associations between increased risks of incident diabetes and SARS-CoV-2 infection were stronger in older (HR=1.292 [95%CI 1.199–1.393], p-interaction<0.001), unvaccinated people (HR=1.694 [95%CI 1.484–1.933], p-interaction<0.001) and people without prediabetes (HR=1.598 [95%CI 1.326–1.926], p-interaction=0.005)*’ has been added to Result section (line 430-434, page 21).

We agree with you that the inclusion criteria requiring an HbA1c measurement could contribute to an apparently stronger association among people without prediabetes. Here are the postulations for the stronger association between increased risks of incident diabetes and SARS-CoV-2 infection among people without prediabetes: (i) people with prediabetes are already predisposed to the development of diabetes such that the additional impact of SARS-CoV-2 infection may be less significant and (ii) people with prediabetes could be more likely to have taken lifestyle modifications to reduce their risk of developing diabetes. The relevant sentences have been added to the Discussion section.

*Relevant text in the Discussion section: “In the subgroup analysis, we also noted an apparently stronger association between increased risks of incident diabetes and SARS-CoV-2 infection among individuals without prediabetes. This could be related to the inclusion criteria requiring an HbA1c measurement before an index date in all individuals. There could be several postulations for this observation: (i) individuals with prediabetes are already predisposed to the development of diabetes such that the additional impact of SARS-CoV-2 infection may be less significant; and (ii) individuals with prediabetes could have more likely taken lifestyle modifications to reduce their risk of developing diabetes.”* *(line 550-556, page 26)*

**As an aside, Table 3 would be easier to read if there were a slight separation, or horizontal lines, between collections of subgroups. Only the overall section of this table needs to be reported. The Type I section shows virtually no data, and the Type II data are almost identical to the overall figures.**

Response R2.4: Thank you. We followed the suggestions from both Reviewer 1 and Reviewer 2. The table has been converted into a forest plot that shows the risks of overall diabetes following COVID-19 vaccination and SARS-CoV-2 infection (Fig 2). The result of type 1 and type 2 diabetes has been removed.

**The authors show that the PS-matching procedure produces well-matched groups, by showing the data and the standardised mean differences. However, they do not report the characteristics of the exposed and unexposed groups prior to matching, except for a few words in the text of the paper, starting at line 256; Table 1 only shows summary data after matching. Perhaps a table could be added to the supplement, showing the full data, and the SMDs, prior to matching?**

Response R2.5: Thank for your suggestions. The baseline characteristics and SMDs before matching have been shown in the S3 Table. The relevant sentences have been added to the Methods and Results sections.

*Relevant text in the Statistical analysis of the Methods section: “Baseline characteristics before and after propensity score matching were presented as means with standard deviation (SD) for continuous variables and frequencies with percentages for categorical variables.” (line 273-275, page 12)*

*Relevant text in the Results section: “In total, we identified 706,631 people in the vaccination first cohort (CoronaVac: n=176,099; BNT162b2: n=182,129; unvaccinated: n=348,403) and 798,578 people in the infection second cohort (COVID-19 patients, n=145,452; non-COVID-19 group, n=653,126) (S3 Table).”* *(line 361-363, page 16)*

**Saying that, in Table 1, the mean ages for the vaccinated groups appear to be about 2 years less than for their matched controls - is this true, or an error in the tables?**

Response R2.6: Thanks for your question. We have checked the numbers in Table 1 and ensured they are correct. The mean ages for vaccinated groups were lower, probably because we did not match the exact age of vaccinated people and their matched controls, and fitted the categorical form (16-44, 45-59, and ≥60 years) to propensity score models. In the paragraph about propensity score matching, we have specified that age was adjusted as a categorical variable in propensity score models (S3 Method).

*Relevant text in the S3 Method: “In the analysis of risks following COVID-19 vaccination, logistic regression model was constructed to estimate the propensity score for each vaccination recipient and control based on the demographic and clinical variables included in previous propensity score weighted cohort study[5-9] and those which were likely to bias the number of incidents reported, including age (categorised into 16-44, 45-59, and ≥60 years), sex, prediabetes[10] (defined as baseline HbA1c ≥5.7% and <6.5%), Charlson comorbidity index (CCI), pre-existing comorbidities, medication use within the 90 days before the index date, and history of SARS-CoV-2 infection.”*

**Reviewer #3: There is still a lack of studies on whether the COVID-19 vaccination has an impact on the incidence of diabetes.**

**This is a well designed, very large study from Hong Kong which shows no increased risk of diabetes after COVID vaccination. Moreover, this study suggests that the diabetes risk after SARS-CoV2 infection is not increased in fully vaccinated persons, and also depends on the type of Corona virus.**
 **I have a few comments on the paper:**

**1. The following publication should also be considered in the discussion:**

**Kwan AC, Ebinger JE, Botting P, Navarrette J, Claggett B, Cheng S. Association of COVID-19 vaccination with risk for incident diabetes after COVID-19 infection. JAMA Netw Open 2023; 6(2):e2255965. doi:10.1001/jamanetworkopen.2022.55965.**

Response R3.1: Thanks for your suggestion. The sentence ‘*Similarly, Kwan et al reported lower diabetes risks after COVID-19 infection in vaccinated than in unvaccinated patients in a study done in the US, suggesting a benefit of vaccination*’ has been added to the Discussion section (line 525-527, page 25).

**2.**  **For BNT162b2, the incidence rate is actually lower for vaccinated than for non-vaccinated persons (cf. that lower risk was also observed by Kwan et al. in the aforementioned paper). The authors do not discuss this but simply state that the incidence is not larger. If you do not think that the decreased risk is causal, what are the reasons for this view?**

Response R3.2: Thanks for your question. The study by Kwan et al. observed a lower risk of incident diabetes following COVID-19 infection among vaccinated persons. In the aforementioned research question, we evaluated the risks of incident diabetes associated with vaccination among people who have not been infected by COVID-19, which differ from the objective of the study by Kwan et al. Our study being observational in nature, we could not firmly establish the causal relationship for the lower risks of incident diabetes after receiving BNT162b2 vaccines. There could be unmeasured confounders which explained the lower risk of incident diabetes for BNT162b2 recipients than non-vaccinated individuals. For instance, individuals who receive the BNT162b2 vaccine were younger and might have also adopted healthier behaviours protecting them from developing diabetes. It is also possible that people with symptoms of undiagnosed diabetes are less likely to get vaccinated due to concerns about vaccine safety or efficacy. This could create a bias in the data that makes it appear as though the vaccine is protective against diabetes when in fact, the reverse is true. More research is needed to fully understand the relationship between the BNT162b2 vaccine and diabetes risk. The relevant sentences have been added to the Discussion section.

*Relevant text in the Discussion section: “For BNT162b2, the incidence rate of diabetes was lower for vaccinated than for unvaccinated individuals. As our study was observational in nature, we could not firmly establish the causal relationship for BNT162b2 lowering the risk of incident diabetes. Unmeasured confounders could have accounted for this apparent protective effect. For instance, BNT162b2 recipients were younger and might have also adopted healthier lifestyle behaviours associated with the reduction in risks of incident diabetes. It is also possible that individuals with symptoms of undiagnosed diabetes were less likely to get vaccinated due to concerns about vaccine safety or efficacy.” (line 472-479, page 23)*

**3. For the control groups, a pseudo-index was assigned. Please give a short explanation in the methods section how this was done.**

Response R3.3: Thanks for your suggestions. We have added the details of the method to assign pseudo-index dates in to Methods section (line 227-230, page 10-11; line 244-248, page 11) with the following sentences ‘*To assign pseudo-index dates for unvaccinated people,* *we matched them with vaccine recipients based on sex and age. To ensure each unvaccinated person could match with a vaccine recipient, the maximum ratio was used. Afterwards, the index date of vaccine recipients was assigned to corresponding unvaccinated people*’ and ‘*To assign pseudo-index dates for people without infection, we matched them with COVID-19 patients based on sex and age. To ensure each non-COVID-19 person could match with a COVID-19 patient, the maximum ratio was used. The index date of COVID-19 patient was then assigned to corresponding non-COVID-19 people*’.

**Minor comments:**
 **4. Page 5, line 96 / 98: Please add the time periods to which the excess risks refer to.**

Response R3.4: Thanks for your suggestion. The median follow-up durations have been added to Introduction section.

*Relevant text in the introduction section: “A cohort using the database from the United States (US) Department of Veteran Affairs showed 40% excess risk of incident diabetes with a median follow-up of one year, mostly type 2 diabetes[5]. Similarly, a study using German nationwide database showed 30% excess risk of incident type 2 diabetes after SARS-CoV-2 infection with a median follow-up duration of 4 months, compared to other acute respiratory illness[6].” (line 137-142, page 7)*

**5. Page 9, line 195: Why do the authors not use ICD-10?**

Response R3.5: Thanks for your question. We used the electronic medical records provided by the Hong Kong Hospital Authority. In electronic medical records, all diagnosed diseases were recorded by ICD-9 codes except for the disease diagnosis coding ‘Cause of Death’ by ICD-10 codes[1]. Therefore, we used ICD-9 to define the diseases in our analysis.

Reference:

1.	Implementation of the Tenth Revision of the International Statistical Classification of Diseases and Related Health Problems. Centre for Health Protection; 2009 [accessed: April 08, 2023]. Available from: <https://www.chp.gov.hk/files/pdf/medcert.pdf>.

**Reviewer #4: The authors used two large cohorts identified by electronic health databases in Hong Kong to evaluate the incidence of diabetes following COVID-19 vaccines and after SARS-CoV-2 infection. For both cohorts, controls were 1:1 matched using propensity scores and HRs for incident diabetes were estimated using Cox regression models. It was found that COVID-19 vaccination was not associated with an increased risk of incident diabetes, while the risk of incident diabetes increased following SARS-CoV-2 infection. This study makes a useful contribution to the literature regarding the incidence of diabetes following COVID-19 vaccination and SARS-CoV-2 infection among the Asian population. I have two major comments which I hope can help the authors further improve the work and its dissemination.**

**1. It will be useful to have a paragraph to briefly introduce the** **COVID-19 pandemic and key policies in Hong Kong, e.g. when did the first patient appear in Hong Kong, when did Hong Kong open up and what's the case number before and after that, what's the vaccination policy (who were prioritized), etc. Such information will help the audience better understand the study design and the interpretation of the results.**

Response R4.1: Thanks for your suggestions. Here is the summary of the COVID-19 pandemic and key policies in Hong Kong. The details of the COVID-19 pandemic and key policies in Hong Kong have been added to S2 Method as follow:

*“The COVID-19 pandemic is a global outbreak of a respiratory illness caused by the novel coronavirus SARS-CoV-2. The outbreak was first identified in Wuhan, China in December 2019 and rapidly spread to other countries, eventually leading to a global pandemic.*

*The first case of COVID-19 in Hong Kong was identified on January 23, 2020, by a tourist from Wuhan. Despite the challenges posed by the pandemic, Hong Kong's response has been relatively successful in controlling the spread of the virus before 2022, with a lower number of confirmed cases. While after 2022, the Omicron variant gradually became the predominant variant (so-called 'fifth wave'), leading to a significant increase in cases and hospitalizations in Hong Kong. The estimated cumulative incidence cases are up to 60.5% of Hong Kong's total population as of March 14, 2022[1]. The fifth wave of the COVID-19 pandemic prompted the government to implement more stringent measures, such as vaccine pass, mandatory mask-wearing and compulsory quarantine, to reduce the flow of people and contain transmission risk in the community. As of August 15, 2022 (the end of the follow-up period in our study), there have been a total of 833,574 confirmed cases of COVID-19 in Hong Kong, with 9,356 deaths[2].*

*Since September 26, 2022, as vaccination rates continued to increase and the number of cases began to decline, the Hong Kong Government lifted the compulsory quarantine requirement for inbound persons from overseas places or Taiwan[3].”*

Here is the summary of the COVID-19 vaccination policy in Hong Kong. The details of the COVID-19 pandemic and key policies in Hong Kong have been added to S1 Method as follow:

*“The mass COVID-19 vaccination program in Hong Kong was started on February 23, 2021 for CoronaVac (inactivated whole-virus vaccine) from Sinovac Biotech (Hong Kong) Limited and March 6, 2021 for BNT162b2 (monovalent mRNA vaccine) from BioNTech/Fosun Pharma in China (equivalent to the Pfizer-BioNTech vaccine outside China). At the very early stage, certain patient groups, like healthcare workers, were prioritised to receive vaccination. However, no guidelines recommend that certain individuals or patient groups receive a particular vaccine or different service delivery. The booking system, used by the Hong Kong Government, automatically schedules two doses of either vaccine for eligible citizens when they register for vaccination using a unique identity document number. Participants can select the preferred vaccine type and switch the vaccine type only after receiving the second dose. The government recommended receiving the third dose at least 90 days following the second dose for the general public not less than 18 years old.*

*As of September 2021, the vaccination uptake rate in Hong Kong was about 60.5% for the first dose (CoronaVac: 22.0%; BNT162b2: 38.5%) and 56.4% for the second dose of COVID-19 vaccine (CoronaVac: 20.5%; BNT162b2: 36.0%). People aged 20-59 years were more likely to receive COVID-19 vaccines, followed by people aged 60 or above. Generally, women are more likely to have received the COVID-19 vaccine than men[4].”*

References:

1.	Further update on estimation of Hong Kong’s infection figures in the fifth wave of COVID-19 D24H2022. Available from: <https://www.d24h.hk/news_event_article/further-update-on-estimation-of-hong-kongs-infection-figures-in-the-fifth-wave-of-covid-19/>.

2.	Archive of Statistics on 5th Wave of COVID-19 The Government of the Hong Kong Special Administrative Region2023 [accessed: April 06, 2023]. Available from: <https://www.coronavirus.gov.hk/eng/5th-wave-statistics.html>.

3.	Press Release: Government announces lifting of compulsory quarantine requirement on arrival at Hong Kong [Internet]. The Government of the Hong Kong Special Administrative Region; 2022 [accessed: April 06, 2023]. Available from: <https://www.info.gov.hk/gia/general/202209/24/P2022092400048.htm?fontSize=1>
4.	COVID-19 Vaccination Programme The Government of the Hong Kong Special Administrative Region; 2023 [accessed: April 02, 2023]. Available from: <https://www.covidvaccine.gov.hk/en/>.

**2. The authors mentioned in the method part that previous SARS-CoV-2 infection was adjusted as a binary variable in the vaccination cohort. How many people had previous SARS-CoV-2 infection in the vaccination cohort? There might be an interaction effect between previous infection and vaccination on the risk of incident diabetes, so it's probably better to drop people who had previous infections in this cohort or conduct a stratification analysis.**
Response R4.2: Thanks for your suggestions. Before propensity score (PS) matching, 459 (0.3%) and 1,210 (0.7%) had previous SARS-CoV-2 infection among CoronaVac recipients and BNT162b2 recipients (S3 Table), respectively. After 1: 1 PS matching, 442 (0.3%) and 693 (0.4%) had previous SARS-CoV-2 infection among CoronaVac recipients and BNT162b2 recipients (Table 1), respectively.

We agree that history of previous infection would modify the association between COVID-19 vaccination and risk of incident diabetes. We have added a subgroup analysis in people with and without previous SARS-CoV-2 infection, the result of these two subgroup analyses were consistent with the main analysis (Fig 2). The relevant sentences have been added to the Methods and Results sections.

*Relevant text in the Methods section: “To evaluate if the association between COVID-19 vaccination and incident diabetes was modified by previous SARS-CoV-2 infection, we performed subgroup analyses by history of SARS-CoV-2 infection.”* *(line 308-311, page 14)*

*Relevant text in the Results section: “We repeated the subgroup analyses according to age, sex, prediabetes, and previous SARS-CoV-2 infection, which showed consistent results for both types of COVID-19 vaccines (Fig 2).”* *(line 401-403, page 19)*

**Reviewer #5: It is a fundamental and quite impressive study assessing the incidence of diabetes after COVID vaccination based on large and representative cohort in HK. I have several comments.
1.Please describe/estimate the prevalence of using the two kinds of vaccinations in HK.**

Response R5.1: Thanks for your suggestion. As of September 2021, the vaccination uptake rate in Hong Kong was about 60.5% for the first dose (CoronaVac: 22.0%; BNT162b2: 38.5%) and 56.4% for the second dose of the COVID-19 vaccine (CoronaVac: 20.5%; BNT162b2: 36.0%). People aged 20-59 years were more likely to receive COVID-19 vaccines, followed by people aged 60 or above. Generally, women are more likely to have received the COVID-19 vaccine than men[1]. The relevant sentences have been added to the Methods. The details of the COVID-19 vaccination policy and vaccine uptake rate in Hong Kong have been added to S1 Method.

*Relevant text in the S1 Method: “As of September 2021, the vaccination uptake rate in Hong Kong was about 60.5% for the first dose (CoronaVac: 22.0%; BNT162b2: 38.5%) and 56.4% for the second dose of COVID-19 vaccine (CoronaVac: 20.4%; BNT162b2: 36.0%). People aged 20-59 years were more likely to receive COVID-19 vaccines, followed by people aged 60 or above. Generally, women are more likely to have received the COVID-19 vaccine than men[1].”*

*Relevant text in the Methods section: “The COVID-19 vaccination policy and vaccine uptake rate in Hong Kong have been elaborated in S1 Method.” (line 223-224, page 10)*

Reference:

1.	COVID-19 Vaccination Programme The Government of the Hong Kong Special Administrative Region; 2023 [accessed: April 02, 2023]. Available from: https://www.covidvaccine.gov.hk/en/.

**2. An individual could belong to both the two cohorts.e.g., one could get vaccination but still be infected. Although you conducted sensitivity analysis to adjust the potential impact, do you also consider to conduct subgroup analysis for the persons who were vaccinated yet infected ?**

Response R5.2: Thanks for your suggestions. We have conducted a subgroup analysis for people who received vaccines and then contracted SARS-CoV-2 infection (Fig 2). The subgroup analysis in our current study stratified by vaccination status revealed a graded attenuation in risk of incident diabetes among COVID-19 survivors compared with their respective non-COVID controls upon completion of COVID-19 vaccination regime (unvaccinated: HR=1.694 [95%CI 1.484–1.933], partially vaccinated: HR=1.204 [95% CI 1.090–1.329], fully vaccinated: HR=1.005 [95%CI 0.904–1.116]).

**3.Do you consider those diagnosed as** **Gestational diabetes? If not, how to separate it from the type I and type II diabetes?**

Response R5.3: Thanks for your question. Gestational diabetes was not included in the original submission, because pregnant women were excluded from the analysis. The inclusion criteria have been revised in the method section.

*Relevant text in the Study design and population of the Methods section: “We identified people (except for pregnant women) who ever used HA services since 2018.” (line 215-216, page 10)*

**4. Line 275-280. BNT162b2 recipients were associated with lower risk, please state this point.**

Response R5.4: Thanks for your question. Our study being observational in nature, we could not firmly establish the causal relationship for the lower risks of incident diabetes after receiving BNT162b2 vaccines. There could be unmeasured confounders which explained the lower risk of incident diabetes for BNT162b2 recipients than non-vaccinated individuals. For instance, individuals who receive the BNT162b2 vaccine were younger and might have also adopted healthier behaviours protecting them from developing diabetes. It is also possible that people with symptoms of undiagnosed diabetes are less likely to get vaccinated due to concerns about vaccine safety or efficacy. This could create a bias in the data that makes it appear as though the vaccine is protective against diabetes when in fact, the reverse is true. More research is needed to fully understand the relationship between the BNT162b2 vaccine and diabetes risk. The relevant sentences have been added to the Discussion section.

*Relevant text in the Discussion section:* *“For BNT162b2, the incidence rate of diabetes was lower for vaccinated than for unvaccinated individuals. As our study was observational in nature, we could not firmly establish the causal relationship for BNT162b2 lowering the risk of incident diabetes. Unmeasured confounders could have accounted for this apparent protective effect. For instance, BNT162b2 recipients were younger and might have also adopted healthier lifestyle behaviours associated with the reduction in risks of incident diabetes. It is also possible that individuals with symptoms of undiagnosed diabetes were less likely to get vaccinated due to concerns about vaccine safety or efficacy.”* *(line 472-479, page 23)*

**5. It seems that the authors did not collect info on diabetic complications, which should be mentioned as a limitation.**

Response R5.5: We have collected the information on the acute diabetic complications such as diabetic ketoacidosis and acute hyperglycemia, and specified this at the Outcome Definition section. The median followed up periods in cohort are relatively short (CoronaVac recipients: 386 days; BNT162b2 recipients: 384 days; COVID-19 patients: 164 days). Given this relatively short follow-up, we could not evaluate the risk of chronic diabetic complication. We have acknowledged it as the limitation in the Discussion section.

*Relevant text in the Outcome of Methods section:* *“In view of case reports of acute hyperglycaemic crises following COVID-19 vaccination, we also retrieved the events of acute hyperglycaemia (ICD-9-CM: 250.82–250.83, 250.20, 250.22–250.23) and diabetic ketoacidosis (DKA) (ICD-9-CM: 250.10, 250.12–250.13 and 250.30–250.33).” (line 266-269, page 12)*

*Relevant text in the Discussion section: “Last but not least, the follow-up period was relatively short in our study to allow evaluation of the risk of chronic diabetic complications.” (line 589-591, page 27)*

We sincerely hope that you will consider this revised manuscript favorably. Should there be further corrections or clarifications needed, we are more than happy to make the necessary changes and provide additional information. We look forward to hearing from you soon.

Yours sincerely,

Carlos King Ho Wong, PhD

Centre for Safe Medication Practice and Research, Department of Pharmacology and Pharmacy, Li Ka Shing Faculty of Medicine, The University of Hong Kong

Rm 1-01, 1/F, Jockey Club Building for Interdisciplinary Research, 5 Sassoon Road, Pokfulam, Hong Kong SAR, China

Phone: (+852) 2831-5055

Fax: (+852) 2814-7475

Email: [carlosho@hku.hk](mailto:carlosho@hku.hk)

Ian Chi Kei Wong, PhD

Centre for Safe Medication Practice and Research, Department of Pharmacology and Pharmacy, Li Ka Shing Faculty of Medicine, The University of Hong Kong

2/F, 21 Sassoon Road, Li Ka Shing Faculty of Medicine, Laboratory Block, Faculty of Medicine Building, Hong Kong SAR, China

Phone: (+852) 3917-9024

Fax: (+852) 2817-0859

Email: [wongick@hku.hk](mailto:wongick@hku.hk)
